# Expression pattern of prohibitin, capping actin protein of muscle Z-line beta subunit and tektin-2 gene in Murrah buffalo sperm and its relationship with sperm motility

**DOI:** 10.5713/ajas.18.0025

**Published:** 2018-04-12

**Authors:** Zhaocheng Xiong, Haihang Zhang, Ben Huang, Qingyou Liu, Yingqun Wang, Deshun Shi, Xiangping Li

**Affiliations:** 1 State Key Laboratory of Conservation and Utilization of Subtropical Agro-bioresources, Guangxi University, Nanning 530004, China; 2 Guangxi Livestock and Poultry Variety Reforming Station, Nanning 530001, China

**Keywords:** Murrah Buffaloes, Prohibitin, Capping Actin Protein of Muscle Z-line Beta Subunit, Tektin-2, Sperm Motility

## Abstract

**Objective:**

The aim of the current study is to investigate the relationship between prohibitin (*PHB*), capping actin protein of muscle Z-line beta subunit (*CAPZB*), and tektin-2 (*TEKT2*) and sperm motility in Murrah buffalo.

**Methods:**

We collected the high-motility and low-motility semen samples, testis, ovary, muscle, kidney, liver, brain and pituitary from Murrah buffalo, and analysed the expression of *PHB*, *CAPZB*, and *TEKT2* in mRNA (message RNA) and protein level.

**Results:**

Quantitative reverse transcription-polymerase chain reaction (qRT-PCR) result showed that the expression of *PHB* was higher and *CAPZB*, *TEKT2* were specifically expressed in testis as compared to the other 6 tissues, and that in testis, the expression of *TEKT2* was higher than that of *CAPZB* and *PHB*. Immunohistochemistry test revealed that all three genes were located on the convoluted seminiferous tubule and enriched in spermatogenic cells. Both qRT-PCR and Western Blot results showed that the expression levels of *PHB*, *CAPZB*, and *TEKT2* were significantly lower in the low-motility semen group compared to the high-motility semen group (p<0.05).

**Conclusion:**

The expression levels of *PHB*, *CAPZB*, and *TEKT2* in Murrah buffalo sperm have a high positive correlation with sperm motility. And the three genes may be potential molecular markers for the decline of buffalo sperm motility.

## INTRODUCTION

Murrah buffalo is an indispensable domestic animal that provides meat, milk, and labor in tropical and subtropical regions. The conception rate and economic benefits of frozen semen production are directly affected by semen quality [1,2]. An important index that measures semen quality is sperm motility. Dungdung et al [3] found that sperm motility regulatory proteins can improve sperm quality [3]. Furthermore, it has been found that motility of sperm is lower in some Murrah buffalo production. Therefore, it is important to investigate the molecular mechanism that underlies the problem of lower motility. Elfateh et al [4] reported that genetic disorders were the most frequent factors causing poor semen quality and decreased sperm motility [4]. Genes including tektin-2 (*TEKT2*) [5], dynein axonemal heavy chain 5 (*DNAH5*), *DNAH11* [6], heat shock protein-70 [7], peroxiredoxin-6 [8], protamine 2 [9] and proteins including vesicle associated membrane protein 4 [10], outer dense fiber protein (*ODF*) [11], a-kinase anchoring protein 4 [12], calcium and integrin binding 1 [13], actin beta (*ACTB*) have been found to be associated with low-motility sperm (asthenospermia) in mice, swine, humans and cattle. However, the molecular mechanism of the low motility of sperm in Murrah buffalo remains unknown. According to our previous study regarding high and low motility of sperm in Murrah buffalo using a differential proteomics method and software analysis, partial protein spots were differentially expressed. The differential protein spot numbers were used to find the corresponding genes in the Uniport database at http://www.uniprot.org/uniprot. We attempted to clone the genes corresponding to the differential protein spots, but could only successfully clone the prohibitin (*PHB*), capping actin protein of muscle Z-line beta subunit (*CAPZB*), and *TEKT2*.

*PHB* is a highly conserved protein that is widely distributed amongst many cell types. It participates in a variety of biological processes such as cell proliferation, differentiation, senescence, and apoptosis [14]. *PHB* has been found in the mitochondria, nucleus and cytoplasm [15]. Currently, it has been reported that *PHB* is related to cell proliferation and mitochondrial function. Previous studies have reported that *CAPZB* is an essential element of the actin cytoskeleton whereby it binds to the barbed ends of actin filaments and regulates their polymerization [16]. Tektins are the constitutive proteins of microtubules in cilia, flagella and centrioles [17]. They were originally isolated from sea urchins as a set of proteins, *tektin A*, *B*, and *C* [18]. Tektins possibly provide stability and structural complexity to axonemal microtubules [19]. Thus, tektins are thought to play a fundamental role in ciliary movement [20].

The cloning and expression patterns of *PHB*, *CAPZB*, and *TEKT2* in Murrah buffalo are seldom reported. Therefore, the three functional genes were cloned in the present study to investigate their expression patterns as possible molecular markers of sperm motility.

## MATERIALS AND METHODS

According to the semen collection records (seen in supplementary materials) of Guangxi Livestock and Poultry Variety Reforming Station and microscopic identification, the semen samples labeled as the high-motility group were obtained from 10 six-year-old Murrah buffalo of which more than sixty percent sperm move forward straightly, the semen samples labeled as the low motility group were obtained from 10 six-year-old Murrah buffalo of which less than sixty percent sperm move forward straightly. In addition, Testis, ovary, muscle, kidney, liver, brain and pituitary from Murrah buffalo were collected from Nanning Luban Road slaughterhouse. And all the samples were stored at −80 degrees for RNA and protein extraction. All procedures were performed according to and approved by the guidelines for the ethical treatment of animals by the Institutional Animal Care and Use Committee of Guangxi University, Nanning, China (Animal Experimental Ethical Inspection Form of Guangxi University was seen in Supplementary materials).

### Cloning and analysis of *PHB*, *CAPZB*, and *TEKT2* genes

Three six-year-old Murrah buffalo testes, ovary, muscle, kidney, liver, brain, and pituitary collected from the local slaughterhouse, and semen samples from high-motility and low-motility Murrah buffaloes were flash frozen, sonicated and crushed, then the total RNA was extracted using the Trizol reagent (Ambion, Life Technologies, New York, USA) according to the manufacturer’s instruction. Three independent preparations were used. According to the bovine *PHB*, *CAPZB*, and *TEKT2* gene sequences in GenBank, the specific primers were designed and synthesized by Shanghai Shengong Biology Co., Ltd. (Shanghai, China) (Table 1). The first-stranded cDNA was synthesized from 2 μg of total RNA from Murrah buffalo testes for reverse transcription-polymerase chain reaction (RT-PCR) by using the Prime Script 1st strand cDNA synthesis kit (Takara, Shiga, Japan). The touchdown PCR was performed to amplify the target fragments. All assessments were conducted in three biological replicates. The PCR products were purified using a TIAN Gen Mini Purification Kit (TIANGEN Biotech; Beijing CO., Ltd, Beijing, China), were inserted into the pMD18-T vector (Takara, Japan), and were transformed into DH 5a E*scherichia coli* (stored in the laboratory). The positive clones were sequenced by the automated sequencing method (BGI-Guangzhou, Guangzhou, China).

The sequencing result of Murrah buffalo *PHB*, *CAPZB*, and *TEKT2* genes was analyzed by NCBI Blast [http://www.ncbi.nlm.nih.gov/BLAST]. The open reading frame (ORF) was predicted by NCBI ORF Finder. Homology analysis was carried out by DNASTAR software. Protein domain, signal peptide, protein subcellular localization and transmembrane structure were respectively predicted by SMART, SignalP 4.1 Server (www.cbs.dtu.dk/services/SignalP), PSORT II (http://psort.hgc.jp/form2.html) and TMHMM 2.0 (http://www.cbs.dtu.dk/services/TMHMM/).

### Analysis of gene expression by quantitative real-time polymerase chain reaction

Quantitative reverse transcription-PCR (qRT-PCR) was performed on the ABI PRISM 7500 Real Time System (Applied Biosystems, Foster City, CA, USA) to determine the expression patterns of *PHB*, *CAPZB*, and *TEKT2* genes by using specific primers as provided in Table 1. The reaction system was 20 μL with 1 μL cDNA, 10 μL 2×FastStart Universal SYBR Green Master, 0.4 μL ROX Reference Dye (Roche, Basel, Switzerland), 0.6 μL primer forward/reverse (10 nmol/L), 8.0 μL RNase-Free H_2_0. Reaction conditions were as follows: 95°C for 10 min; 95°C for 15 s, 60°C for 1 minute for 40 cycles. Each sample was repeated 3 times. *ACTB* was used as the reference gene. The 2^−^^ΔΔ^^CT^ method was used to calculate the relative expression of each gene.

### Immunohistochemistry

Murrah buffalo testis was fixed in 4% paraformaldehyde (Sigma, Saint Louis, MO, USA), dehydrated and paraffin embedded. The 5-μm-thick serial sections were obtained by Leica RM 2235 rotary microtome. The sections were processed in APES-acetone (1:49), de-paraffinized and rehydrated. Then, the sections were incubated with 3% hydrogen peroxide in methanol, boiled in 10 mM sodium citrate buffer, and permeabilized in 1% Triton X-100. After blocking with 5% bovine serum albumin, the sections were incubated with the primary antibody (*PHB* at 1:100 dilution, GeneTex, Irvine, CA, USA, GTX124491; *CAPZB* at 1:100 dilution, GeneTex, GTX101686; *TEKT2* at 1:50 dilution, Proteintech 13518-1-AP, Chicago, IL, USA) overnight at 4°C. Sections were then washed 3 times in phosphate buffer saline (PBS)-Tween-20, incubated with Biotin-labeled goat anti-rabbit IgG at 1:100 dilution, (Proteintech SA00001-2, USA) for 45 minutes at room temperature and then at 45 minutes at 37°C. Immunoreactive signal was detected using streptavidin-HRP and diaminobenzidine (DAB Map Kit, Ventana, Tucson, AZ, USA). The negative controls were generated by replacing the primary antibody with PBS. The sections were observed with an Olympus DP70 digital camera mounted on a Leica DMR microscope with Nomarski optics (Leica, Heerbrugg, Switzerland).

### Sperm microstructure observation

Sperm motility means the ratio of sperm moving forward in a straight line, which is one of the main standards evaluating the semen quality. All sperms moving forward in a line can get a score of 1.0, 90% sperms moving forward in a line can get a score of 0.9, and so on, and fresh sperm is qualified when its motility reaches no less than 0.6 [21]. According to the method of making optical microscope specimen of Guangxi Livestock and Poultry Variety Reforming Station, Murrah buffalo semen was diluted with 2.9% sodium citrate and dropped onto the glass slide. Semen was classified into two groups according to whether the ratio of sperm moving forward straightly was above 60% under optical microscope at 38°C to 40°C (400×), and the group equal or greater than 60% was named high-motility sperm, while the group below 60% was named low-motility sperm.

### Western-blot analysis

Murrah buffalo semen of high- and low-motility sperm were centrifuged at 700×g for 15 min to remove seminal plasma, washed and centrifuged at 700×g for 15 min three times in PBS, and split with cell lysis buffer radio-immunoprecipitation assay (RIPA) and protease inhibitor phenylmethanesulfonyl fluoride on ice for 30 minutes before Western Blot analysis of the sperm samples was performed according to standard protocol. Briefly, the sperm samples were lysed in RIPA buffer with 0.1% cocktail and 10% phosphotransferase inhibitor. Then the samples were separated by sodium dodecyl sulfate–polyacrylamide gel electrophoresis and transferred on nitrocellulose membranes (BIO-RAD Membrane, Hercules, CA, USA; 0.22 μm) by semidry transfer cell (BIO-RAD, USA). The membranes were blocked in tris-HCl buffer (TBS) containing 0.1% Tween 20 (TBST) containing 5% nonfat milk at room temperature for 45 min. After washing three times in TBST for 10 min each, followed by adding either of the following primary antibodies (*PHB* at dilution of 1:500, GeneTex GTX124491, USA; *CAPZB* at dilution of 1:500, GeneTex GTX101686, USA; *TEKT2* at dilution of 1:500, Proteintech 13518-1-AP, USA; *ACTB* at dilution of 1:1,000, Cell Signaling Technology, Danvers, MA, USA) overnight at 4°C. The blots were then incubated with the second antibody (at dilution of 1:1,000, Proteintech SA0000 1-2, USA) for 1 hour at room temperature. The membranes were washed 3 times in TBST and processed for detection by electro-chemi-luminescence with the Bio-Rad ChemiDoc system (USA). Gray level of Western blot band was quantified with the image J analyzer, *ACTB* was made as the reference, the gray level of high-motility sperm group was normalized, and then calculated the relative expression level of the low-motility sperm group.

### Statistical analysis

Data were analyzed by one-way analysis of variance. The results were compared using the least-significant difference method in the SPSS statistical package 16.0 (SPSS Inc., Chicago, IL, USA). The p values less than (<) 0.05 were considered significant.

## RESULTS

### Cloning of Murrah buffalo *PHB*, *CAPZB*, and *TEKT2* genes and bioinformatics analysis

**Figure 1:**
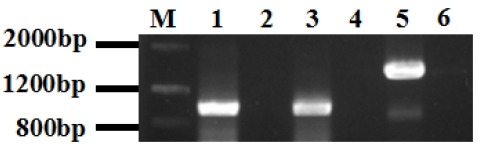
The amplification results of Murrah buffalo *PHB*, *CAPZB*, *TEKT2* coding sequnence (CDS). *PHB*, prohibitin; *CAPZB*, capping actin protein of muscle Z-line beta subunit; *TEKT2*, tektin-2; PCR, polymerase chain reaction. M, Marker III; 1, PCR product of Murrah buffalo *PHB*, 948 bp; 3, PCR product of Murrah buffalo *CAPZB*, 958 bp; 5, PCR product of Murrah buffalo *TEKT2*, 1,449-bp; 2,4,6, negative control.

In order to determine the expression patterns of *PHB*, *CAPZB*, and *TEKT2* genes in Murrah buffalo tissues and sperm, cDNA fragments of 948-bp, 958-bp, and 1,449-bp for *PHB*, *CAPZB*, and *TEKT2* respectively were amplified and sequenced from the RNA samples of Murrah buffalo testes (Supplementary Table S1). It contained an open reading frame of 819 bp, 906 bp, and 1,293 bp, respectively, encoding a protein of 272, 301, and 430 amino acids, respectively (Figure 1). Homology analysis results revealed that Murrah buffalo *PHB*, *CAPZB*, and *TEKT2* genes had more than 90% similarity with the nucleotide sequences of *Bos taurus*, *Sus scrofa*, *Ovis aries*, *Pan troglodytes*, and *Homo sapiens*. Murrah buffalo *CAPZB* had 100% similarity with amino acid sequences of *Bos taurus*, *Sus scrofa*, *Pan troglodytes*, and *Homo sapiens* and 99% similarity with *Ovis aries* and *Mus musculus*. These findings suggest high conservation during evolution. Murrah buffalo *PHB* and *TEKT2* amino acid sequences were also highly conserved in different species (Tables 2, 3), such as Cattle (*Bos taurus*), wild boar (*Sus scrofa*), sheep (*Ovis aries*), chimpanzee (*Pan troglodytes*), human being (*Homo sapiens*) and mosue (*Mus musculus*). The 3 genes did not contain a signal peptide sequence. The results of cell electronic location showed that Murrah buffalo *PHB*, *CAPZB*, and *TEKT2* were possibly located in the mitochondria, the cytoskeleton and the cytoplasm, respectively. *PHB* protein was predicted to contain a transmembrane structure and one domain. *CAPZB* and *TEKT2* proteins were not predicted to have these characteristics.

### Expression of *PHB*, *CAPZB*, and *TEKT2* genes in Murrah buffalo tissues

**Figure 2:**
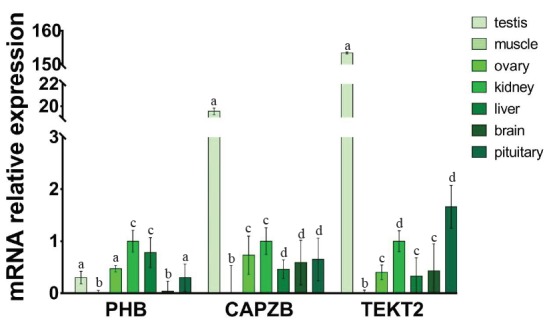
*PHB* (a), *CAPZB* (b), *TEKT2* (c) mRNA expression in different tissues of Murrah buffalo. *PHB*, prohibitin; *CAPZB*, capping actin protein of muscle Z-line beta subunit; *TEKT2*, tektin-2. Data represent mean±standard deviation of three replicates, and the different superscripts in the same table indicate significant difference (p<0.05).

**Figure 3:**
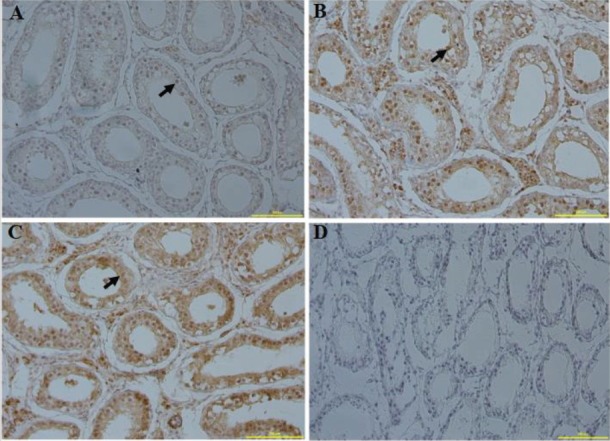
The immunohistochemical staining of tissue section from Murrah buffalo testis for the localization of *PHB* (a), *CAPZB* (b), *TEKT2* (c) and testis negative control (d). *PHB*, prohibitin; *CAPZB*, capping actin protein of muscle Z-line beta subunit; *TEKT2*, tektin-2. Bar, 100 μm.

To investigate the expression patterns of *PHB*, *CAPZB*, and *TEKT2* genes in different tissues, the relative expression levels of *PHB*, *CAPZB*, and *TEKT2* were analyzed by qRT-PCR. As shown in Figure 2, *PHB* gene has a high expression in the testis, the kidney, the liver, the ovary and the pituitary gland, while Low expression in the brain and minimal expression in the muscle. Expression of the *CAPZB* gene in the testis was significantly higher than that in the kidney, the ovary, the pituitary gland and the brain (p<0.05). The expression of the *TEKT2* gene had the most abundant expression in the testis as compared to its levels in the kidney, the liver, the ovary, the pituitary gland and the brain. There was almost no expression of *TETKT2* in muscle (p<0.01). Because *PHB*, *CAPZB*, and *TEKT2* genes were highly expressed in the testis, we further assayed its protein levels using immunohistochemistry. As shown in Figure 3, *PHB*, *CAPZB*, and *TEKT2* proteins were expressed in the testis with the expression of *TEKT2* higher than *CAPZB* and *PHB*. The expression of *PHB* was the lowest among the 3 proteins, which is consistent with the qRT-PCR result showing the mRNA expression of *PHB* as the lowest amongst the 3 genes in the testis. *PHB*, *CAPZB*, and *TEKT2* proteins were located on the convoluted seminiferous tubule and enriched in spermatogenic cells of different developmental stages (Figure 3).

### Microstructure of the high- and low-motility sperm samples

**Figure 4:**
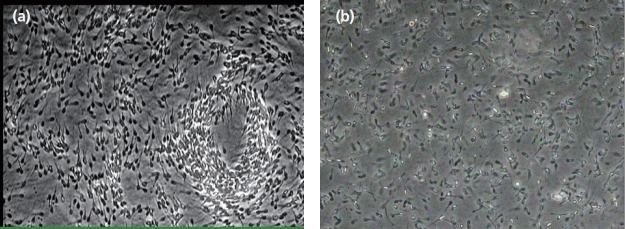
High- and low-motility sperm morphology under optical microscope. The sperm sample with high motility (a) and the sperm sample with low motility (b). Sperm Motility means the ratio of sperm moving forward in a straight line. (a) 400x; (b) 400x.

Under optical microscope, the ratio of sperm moving forward in a straight line in high-motility sperm sample was 74%, whereas in the low-motility sperm sample, the ratio was 37%. This difference in motility between the two groups was significant (p<0.05) (Table 4). The high-motility sperm sample showed a cloud shape, with a stream of sperm that moved forward in a straight line. In the low-motility sperm sample, dead sperm and deformed sperm were observed with only a few sperms moving forward in a straight line (Figure 4A, 4B).

### Expression patterns of *PHB*, *CAPZB*, and *TEKT2* in sperm

**Figure 5:**
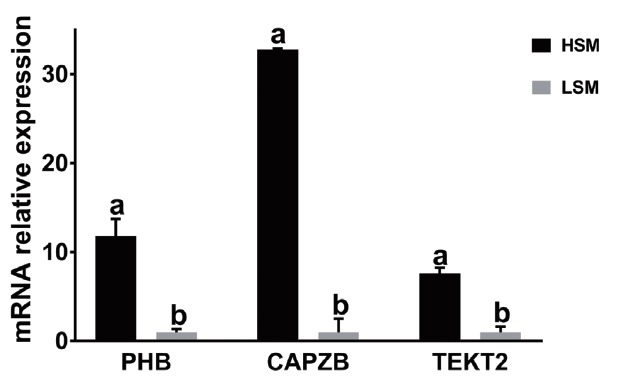
*PHB* (a), *CAPZB* (b), and *TEKT2* (c) mRNA expression in the sperm samples with high and low motility. “HSM” represents the sperm sample with high motility while. “LSM” represents the sperm sample with low motility. *PHB*, prohibitin; *CAPZB*, capping actin protein of muscle Z-line beta subunit; *TEKT2*, tektin-2. Data represent mean±standard deviation of three replicates, and the different superscripts in the same table indicate significant difference (p<0.05).

**Figure 6:**
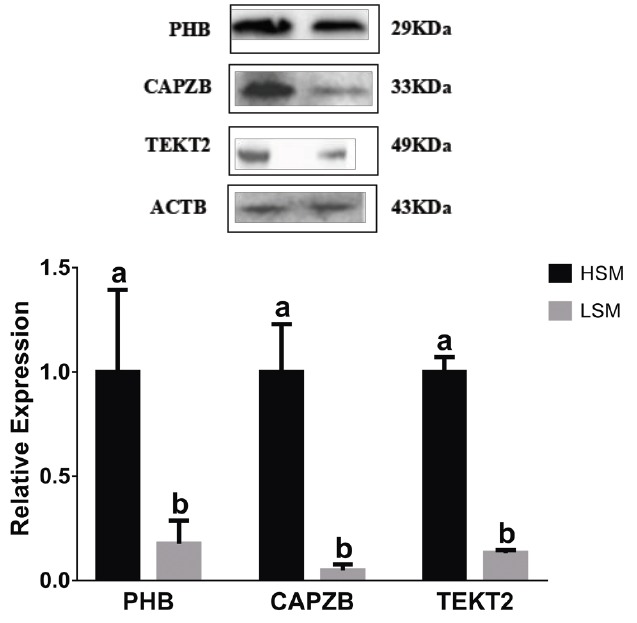
*PHB*, *CAPZB*, and *TEKT2* protein expression in the sperm samples with high and low motility. 1, the sperm sample with high motility; 2, the sperm sample with low motility. *PHB*, prohibitin; *CAPZB*, capping actin protein of muscle Z-line beta subunit; *TEKT2*, tektin-2. Data represent mean±standard deviation of three replicates, and the different superscripts in the same table indicate significant difference (p<0.05).

To study the relationship between the expression and sperm motility, the expression levels of *PHB*, *CAPZB*, and *TEKT2* in sperm were analyzed by qRT-PCR and Western blot. As shown in Tables 4 and Figure 5, the relative mRNA expressions of *PHB*, *CAPZB*, and *TEKT2* were lower in the low-motility sperm group than those in high-motility sperm group. The difference between the high-motility sperm group and the low-motility sperm group was significant (p<0.05). As shown in Figure 6, proteins expression of *PHB*, *CAPZB*, and *TEKT2* were significantly lower in the low-motility sperm group than those in the high-motility sperm group (p<0.05).

## DISCUSSION

In recent years, some progresses have been made on the study of low sperm-motility sperm in human, mice and so on. Large-scale proteomic studies provided protein biomarkers associated with sperm quality [22–27]. Yu-lin Huang et al. found 18 different expression protein spots (four of them were successfully identified by Mass Spectrometry as belonging to three unique proteins- *ODF2*, ATP synthase F1 subunit alpha pseudogene 1, and succinate-CoA ligase GDP-forming beta subunit, which are related to energy metabolism) between high- and low-motility Murrah buffalo sperm by using comparative proteomics [28]. Kwon WS found that in the capacitated spermatozoa, the expression of cytochrome b-c1 complex subunit 1 and ras-related protein Rab-2A negatively correlated with litter size, while the expression of cytochrome b-c1 complex subunit 2 positively correlated with litter size, and these genes may be used as markers for prognosis and diagnosis of male fertility [29,30]. Moreover, it has been reported that the expressions of some genes (e.g. enolase 1, ATP synthase H+ transporting mitochondrial F1 complex beta subunit, etc.) in sperm of low-motility were significantly lower and some other genes (voltage dependent anion channel 2, ropporin-1, etc.) were significantly higher than that in sperm of high-motility [31], but there are no reports in Murrah buffaloes. In the present study, *PHB*, *CAPZB*, and *TEKT2* were cloned and analyzed for the first time. Homology analysis has shown that *PHB*, *CAPZB*, and *TEKT2* are highly conserved among Murrah buffalo and other species. We explored the relationship between the expressions of *PHB*, *CAPZB*, and *TEKT2* in sperm and sperm motility. The relative expressions of *PHB*, *CAPZB*, and *TEKT2* mRNA were down-regulated in the low-motility sperm group compared to the high-motility group.

*PHB* is a highly conserved membrane protein of mitochondria in sperm. It has been reported that *PHB* plays an important role in maintaining mitochondrial structure and function as a new kind of molecular chaperone. In addition, the natural substrates of *PHB* in mitochondria include cytochrome oxidase and mitochondrial oxydation respiratory chain complex I. *PHB* adjusts their assembly and degradation. In addition, it also can shift lytic membrane protein matrix out of mitochondrion. In nematode, the loss of *PHB* leads to biogenous defect of mitochondria, which suggests that *PHB* is crucial to maintain normal development of the mitochondria [32]. In mammalian fibroblast and yeast, the expression of *PHB* changed with cell aging, which suggests that the down-regulation of *PHB* expression is closely related to the accumulation of oxygen free radicals in mitochondrion [33,34]. In the present study, we detected the mRNA and protein expression of *PHB* in sperm. We found that the expression of *PHB* mRNA and protein in the low-motility sperm group was significantly lower than that in the high-motility sperm group. This suggests that the change of *PHB* expression may affect sperm motility. *PHB* may play a role in regulating mitochondrial respiration activity and aging.

*CAPZB* is known to increase actin filament depolymerization and capping, which promotes cell motility [35]. Several previous reports have briefly described the functions of *CAPZB* [36–38], focusing on its role as a capping protein (*CP*). *CPs* are important for the dynamics of actin filament assembly and regulation of the cell shape and movement [39,40]. In the present study, we detected the mRNA and protein expression of *CAPZB* gene in sperm. We found that the expression of *CAPZB* mRNA and protein in the low-motility sperm group was significantly lower than that in the high-motility sperm group. This suggests that the expression of *CAPZB* may be related to sperm motility. *CAPZB* may play a role in regulation of cell morphology and cytoskeletal organization.

*Tektins* are evolutionarily conserved filament-forming proteins localized in flagella and cilia that have been reported to be involved in the stability and structural complexity of axonemal microtubules. Five mammalian *Tektins* (*Tektin1* through 5) have been reported. Of these, *TEKT2* has been found to be required for normal flagellum structure and function. *TEKT2*-null sperm has displayed flagellum bend and reduced motility, probably due to disruption of the dynein inner arm [41]. Confocal laser scanning microscopy and pre-embedding immunoelectron microscopy have revealed that *TEKT2* is associated with the surface of *ODFs*. *TEKT2* may function as an *ODF*-affiliated molecule required for flagellum stability and sperm motility [42]. In the present study, we detected the expression of *TEKT2* gene and protein in sperm. We found that the expression of *TEKT2* mRNA and protein in the low-motility sperm group was significantly lower than that in the high-motility sperm group. This suggests that the change of *TEKT2* expression may affect sperm motility. *TEKT2* may play a role in assembly and attachment of inner dynein arm in sperm flagella.

## CONCLUSION

In summary, our results suggest that *PHB*, *CAPZB*, and *TEKT2* are highly conserved among different species. The expression levels of *PHB*, *CAPZB*, and *TEKT2* in Murrah buffalo sperm have a high positive correlation with sperm motility. And the three genes may be potential molecular markers for the decline of buffalo sperm motility.

## Figures and Tables

**Table 1:** Primers for RT-PCR and qRT-PCR

No.	Buffalo gene name	Sequence (5′-3′)	Product (bp)	Annealing temperature (°C)	Remarks
1	*PHB*	Up: 5′-GTCCAGCCAAAGGAGACA-3′ Lw: 5′-CCTTCACTTTAAGCCAATCA-3′	948	60.0	RT-PCR
2	*CAPZB*	Up: 5′-CTCCGAGGCCAGCAGA-3′ Lw: 5′-CGGTCAGTGGGAAGCA-3′	958	60.7	RT-PCR
3	*TEKT2*	Up: 5′-GCGGAAGGAGAGTTAGGTG -3′ Lw:5′-ACTGAGAATCCCGACCTTTA-3′	1,449	61.0	RT-PCR
4	*PHB*	Up: 5′-GCTGCCATCCATCACTACA-3′ Lw: 5′-GTCATCGCTCACCTGTCTG-3′	106	61.0	qRT-PCR
5	*CAPZB*	Up: 5′-CAATGGGCTGAGGTCTGT-3′ Lw: 5′-AGCAGGAAACGAGTGTCTAAG-3′	172	60.5	qRT-PCR
6	*TEKT2*	Up: 5′-ACCCGCACTACAAACCG -3′ Lw:5′-GGACTGAGAATCCCGACCTT-3′	156	61.0	qRT-PCR
7	*ACTB*	Up: 5′-ACCGCAAATGCTTCTAGG-3′ Lw: 5′-ATCCAACCGACTGCTGTC-3′	182	58	qRT-PCR

*PHB*, prohibitin; RT-PCR, reverse transcription polymerase chain reaction; *CAPZB*, capping actin protein of muscle Z-line beta subunit; qRT-PCR, quantitative reverse transcription-polymerase chain reaction; *TEKT2*, tektin-2; *ACTB*, actin beta.

**Table 2:** The homologous analysis of Murrah buffalo *PHB*, *CAPZB*, and *TEKT2* DNA sequences

Items	*Bos taurus* (%)	*Sus scrofa* (%)	*Ovis aries* (%)	*Pan troglodytes* (%)	*Homo sapiens* (%)	*Mus musculus* (%)
*PHB*	99.4	94.3	97.8	92.8	92.9	89.6
*CAPZB*	99.3	94.3	97.6	92.9	93.2	90.2
*TEKT2*	98.4	92.3	96.6	90.3	90.2	83.7

*PHB*, prohibitin; *CAPZB*, capping actin protein of muscle Z-line beta subunit; *TEKT2*, tektin-2.

**Table 3:** The homologous analysis of Murrah buffalo *PHB*, *CAPZB*, and *TEKT2* amino acid sequences

Items	*Bos taurus* (%)	*Sus scrofa* (%)	*Ovis aries* (%)	*Pan troglodytes* (%)	*Homo sapiens* (%)	*Mus musculus* (%)
*PHB*	99.6	97.1	99.6	99.6	99.6	99.3
*CAPZB*	100	100	99	100	100	99
*TEKT2*	98.1	93.3	96.1	91.0	90.7	84

*PHB*, prohibitin; *CAPZB*, capping actin protein of muscle Z-line beta subunit; *TEKT2*, tektin-2.

**Table 4:** The sperm samples with high and low motility main parameter comparison1)

Sample	Quantity (mL)	Density (10^9^/mL)	Sperm motility
HSM	7.7±2.04^a^	7.40±1.01^a^	0.74±0.01^a^
LSM	6.7±0.81^b^	5.49±0.76^b^	0.37±0.09^b^

HSM, the sperm sample with high motility; LSM, the sperm sample with low motility.

1) n = 10, mean±standard error.

ab The different superscripts in the same table indicate significant difference (p<0.05).

## References

[1] Park YJ, Kim J, You YA, Pang MG (2013). Proteomic revolution to improve tools for evaluating male fertility in animals. J Proteome Res.

[2] Rahman MS, Lee JS, Kwon WS, Pang MG (2013). Sperm proteomics: road to male fertility and contraception. Int J Endocrinol.

[3] Dungdung SR, Bhoumik A, Saha S, Carreira RP (2016). Sperm motility regulatory proteins: a tool to enhance sperm quality. Insights from animal reproduction.

[4] Elfateh F, Wang R, Zhang Z, Jiang Y, Chen S, Liu R (2014). Influence of genetic abnormalities on semen quality and male fertility: A four-year prospective study. Iran J Reprod Med.

[5] Tanaka H, Iguchi N, Toyama Y (2004). Mice deficient in the axonemal protein *Tektin-t* exhibit male infertility and immotile-cilium syndrome due to impaired inner arm dynein function. Mol Cell Biol.

[6] Zuccarello D, Ferlin A, Cazzadore C (2008). Mutations in dynein genes in patients affected by isolated non-syndromic asthenozoospermia. Hum Reprod.

[7] Huang SY, Chen MY, Lin EC (2002). Effects of single nucleotide polymorphisms in the 5′-flanking region of heat shock protein 70.2 gene on semen quality in boars. Anim Reprod Sci.

[8] Ambruso DR, Ellison MA, Thurman GW, Leto DR (2012). Peroxiredoxin 6 translocates to the plasma membrane during neutrophil activation and is required for optimal *NADPH oxidase* activity. Biochim Biophys Acta.

[9] Steger K, Wilhelm J, Konrad L (2008). Both protamine-1 to protamine-2 mRNA ratio and *Bcl2* mRNA content in testicular spermatids and ejaculated spermatozoa discriminate between fertile and infertile men. Hum Reprod.

[10] Williams D, Pessin JE (2008). Mapping of *R-SNARE* function at distinct intracellular *GLUT4* trafficking steps in adipocytes. J Cell Biol.

[11] Amaral A, Cast illo J, Ramalho-Santos J, Oliva R (2014). The combined human sperm proteome: cellular pathways and implications for basic and clinical science. Hum Reprod Update.

[12] Baccetti B, Collodel G, Estenoz M (2005). Gene deletions in an infertile man with sperm fibrous sheath dysplasia. Hum Reprod.

[13] Yu Y, Song X, Du L, Wang C (2009). Molecular characterization of the sheep *CIB1* gene. Mol Biol Rep.

[14] Mishra S, Murphy LC, Murphy LJ (2006). The *prohibitins*: emerging roles in diverse functions. J Cell Mol Med.

[15] Merkwirth C, Langer T (2009). *Prohibitin* function within mitochondria: essential roles for cell proliferation and cristae morphogenesis. Biochim Biophys Acta.

[16] Cooper JA, Sept D (2008). New insights into mechanism and regulation of actin capping protein. Int Rev Cell Mol Biol.

[17] Steffen W, Linck RW (1988). Evidence for *tektins* in centrioles and axonemal microtubules. Proc Natl Acad Sci USA.

[18] Norrander JM, Perrone CA, Amos LA, Linck RW (1996). Structural comparison of tektins and evidence for their determination of complex spacings in flagellar microtubules. J Mol Biol.

[19] Linck RW, Stephens RE (1987). Biochemical characterization of *tektins* from sperm flagellar doublet microtubules. J Cell Biol.

[20] Nojima D, Linck RW, Egelman EH (1995). At least one of the protofilaments in flagellar microtubules is not composed of tubulin. Curr Biol.

[21] Zhang CX, Wu WC, Zou LS, Shi RX (2000). Science and technology in Chinese buffaloes.

[22] Park YJ, Kim J, You YA, Pang MG (2013). Proteomic revolution to improve tools for evaluating male fertility in animals. J Proteome Res.

[23] Rahman MS, Lee JS, Kwon WS, Pang MG (2013). Sperm proteomics: road to male fertility and contraception. Int J Endocrinol.

[24] Kwon WS, Rahman MS, Ryu DY, Khatun A, Pang MG (2017). Comparison of markers predicting litter size in different pig breeds. Andrology.

[25] Rahman MS, Kwon WS, Pang MG (2017). Prediction of male fertility using capacitation-associated proteins in spermatozoa. Mol Reprod Dev.

[26] Yoon SJ, Rahman MS, Kwon WS (2016). Proteomic identification of cryostress in epididymal spermatozoa. J Anim Sci Biotechnol.

[27] Kwon WS, Rahman MS, Lee JS (2015). Discovery of predictive biomarkers for litter size in boar spermatozoa. Mol Cell Proteomics.

[28] Huang YL, Fu Q, Yang L (2015). Differences between high-and low-motility Mmurrah buffalo sperm identified by comparative proteomics. Reprod Domest Anim.

[29] Kwon WS, Rahman MS, Ryu DY, Park YJ, Pang MG (2015). Increased male fertility using fertility-related biomarkers. Sci Rep.

[30] Kwon WS, Oh SA, Kim YJ (2015). Proteomic approaches for profiling negative fertility markers in inferior boar spermatozoa. Sci Rep.

[31] Park YJ, Kwon WS, Oh SA, Pang MG (2012). Fertility-related proteomic profiling bull spermatozoa separated by percoll. J Proteome Res.

[32] Sharma A, Qadri A (2004). Vi polysaccharide of Salmonella typhi targets the prohibitin family of molecules in intestinal epithelial cells and suppresses early inflammatory responses. Proc Natl Acad Sci USA.

[33] Roskams AJ, Friedman V, Wood CM (1993). Cell cycle activity and expression of prohibitin mRNA. Cell Physiol.

[34] Piper PW, Jones GW, Bringloe D (2002). The shortened replieative lifespan of prohibitin mutants of yeast appears to be due to defective mitochondrial segregation in old mother cells. Aging Cell.

[35] Bai SW, Herrera-Abreu MT, Rohn JL (2011). Identification and characterization of a set of conserved and new regulators of cytoskeletal organization, cell morphology and migration. BMC Biol.

[36] Shimada K, Uzawa K, Kato M (2005). Aberrant expression of *RAB1A* in human tongue cancer. Br J Cancer.

[37] Vignjevic D, Montagnac G (2008). Reorganisation of the dendritic actin network during cancer cell migration and invasion. Semin Cancer Biol.

[38] Carlier MF, Pantaloni D (1997). Control of actin dynamics in cell motility. J Mol Biol.

[39] Hopmann R, Cooper JA, Miller KG (1996). Actin organization, bristle morphology, and viability are affected by actin capping protein mutations in Drosophila. J Cell Biol.

[40] Cooper JA, Sept D (2008). New insights into mechanism and regulation of actin capping protein. Int Rev Cell Mol Biol.

[41] Tanaka H, Iguchi N, Toyama Y (2004). Mice deficient in the axonemal protein *Tektin-t* exhibit male infertility and immotile-cilium syndrome due to impaired inner arm dynein function. Mol Cell Biol.

[42] Shimasaki S, Yamamoto E, Murayama E (2010). Subcellular localization of *Tektin2* in rat sperm flagellum. Zoolog Sci.

