# Supplementary material for: Expression pattern of prohibitin, capping actin protein of muscle Z-line beta subunit and tektin-2 gene in Murrah buffalo sperm and its relationship with sperm motility
Source: Asian-Australas J Anim Sci. 2018 Apr 12;31(11):1729–37. doi: 10.5713/ajas.18.0025 (PMC6212766; doi:10.5713/ajas.18.0025)
Supplement: Supplementary file 1 [file ajas-31-11-1729-supplementary.pdf]

Supplementary Materials

2014 high motility sperm record

Species: Murrah buffalo   Buffalo NO.: 1

Date(m/d/y)	Room temperature	Original semen			Diluted				Motility after frozen
		Quantity (ml)	Sperm Motility	Density(10^9/ml)	Quantity of dilution buffer	Actual volume of addition	Total volume	equilibrium time	
1/2/2014	20	7.1	0.76	3.81	9	15	15.8	10:38	0.34
1/6/2014	19	6.2	0.70	3.92	8	10	10.7	10:15	0.34
1/9/2014	13	8.6	0.65	3.74	11	12	12.7	10:02	0.30
1/13/2014	10	8.0	0.72	7.52	28	28	28.7	10:09	0.32
1/16/2014	8	7.1	0.75	3.98	10	12	12.8	9:32	0.35
1/20/2014	9	7.2	0.70	2.49	4	5	5.7	9:31	0.36
1/23/2014	6	9.1	0.65	4.09	13	14	14.7	9:42	0.35
1/27/2014	18	10.8	0.68	3.22	10	10	10.7	9:28	0.33
2/13/2014	6	8.3	0.65	3.12	7	12	12.7	9:31	0.32
2/17/2014	11	10.9	0.65	1.76	1	7	7.7	9:46	0.33
2/20/2014	4	3.9	0.70	2.78	3	3	3.7	10:11	0.35
2/24/2014	13	7.0	0.70	5.04	14	15	15.7	10:19	0.33
2/27/2014	16	9.2	0.75	3.76	12	14	14.8	9:49	0.31
3/3/2014	13	8.7	0.65	3.83	11	11	11.7	9:50	0.33
3/6/2014	14	9.9	0.76	3.42	10	10	10.8	9:24	0.35
3/10/2014	11	8.3	0.75	2.79	6	15	15.8	9:54	0.36
3/13/2014	16	7.1	0.69	8.10	27	27	27.7		0.34
3/17/2014	15	10.0	0.73	7.50	35	30	30.7	9:55	0.32
3/20/2014	22	5.2	0.70	3.17	5	5	5.7	9:56	0.35
3/24/2014	21	5.0	0.72	2.28	2		0.7		0.30
3/27/2014	21	4.5	0.73	7.30	15	15	15.7	10:57	0.32
3/31/2014	24	7.1	0.73	5.71	17	17	17.7	10:09	0.38
4/7/2014	21	7.3	0.65	2.15	2	6	6.7	10:50	0.30
4/10/2014	22	11.1	0.65	3.18	10	10	10.7	10:09	0.30
4/14/2014	25	8.5	0.70	5.66	20	20	20.7	10:00	0.35
4/17/2014	25	4.8	0.76	6.38	14	15	15.8	10:11	0.32
4/21/2014	27	13.4	0.65	1.92	2	5	5.7	10:01	0.10
4/24/2014	25	6.3	0.70	2.60	2	2	2.7	10:01	0.34

4/28/2014	26	10.8	0.60	1.37	2	2	2.6	10:06	0.30	
5/5/2014	20	13.4	0.75	7.15	44	43	43.8	10:07	0.20	
5/8/2014	22	8.5	0.75	6.39	24	24	24.8	10:11	0.32	
5/19/2014	27	11.5	0.75	9.21	52	50	50.8	9:40	0.33	
5/22/2014	29	7.3	0.70	7.59	26	25	25.7	9:45	0.40	
5/26/2014	31	9.8	0.76	9.21	44	45	45.8	9:40	0.40	
6/5/2014	30	18.4	0.65	3.98	26	25	25.7	9:44	0.32	
6/9/2014	27	9.9	0.70	2.18	3	18	18.7	10:09	0.40	
6/16/2014	30	8.2	0.70	4.64		15	15.7	10:25	0.38	
6/23/2014	30	12.5	0.76	6.68	38	53	53.8	9:54	0.35	
6/30/2014	30	7.9	0.76	7.49	28	29	29.8	10:10	0.32	
7/7/2014	30	13.6	0.75	5.95	35	35	35.8	9:27	0.33	
7/14/2014	30	12.8	0.69	3.48	14	13	13.7	9:44	0.35	
7/21/2014	28	6.7	0.75	5.99	17	17	17.8	9:29	0.36	
7/28/2014	30	10.6	0.70	3.37	11	11	11.7	9:20	0.32	
8/4/2014	31	12.2	0.65	3.73	15	15	27.2	9:26	0.31	
8/11/2014	31	14.9	0.68	2.42	7	15	29.9	9:17	0.30	
9/11/2014	29	10.5	0.77	6.70	32	31	42	9:10	0.33	
9/15/2014	31	10.6	0.70	3.64	13	15	26	9:25	0.32	
9/22/2014	29	6.9	0.65	2.82	5	7	14	9:30	0.30	
9/29/2014	28	14.9	0.70	3.22	20	40	55	9:30	0.40	
10/9/2014	22	6.9	0.75	2.55	4	7	7.8	9:49	0.30	
10/13/2014	24	8.2	0.70	3.74	10	12	12.7	8:30	0.35	
10/16/2014	22	8.5	0.70	3.80	11	13	13.7	8:56	0.34	
10/20/2014	27	9.0	0.76	6.55	26	30	30.8	9:31	0.34	
10/27/2014	26	8.0	0.70	4.31	13	15	15.7	9:47	0.36	
11/6/2014	20	13.0	0.76	5.19	28	39	52	9：35	0.45	
11/10/2014	18	7.7	0.65	3.43	8	10	18	9.41	0.40	
11/17/2014	17	13.4	0.65	1.25	3	10	23	9:39	0.35	
11/24/2014	19	9.5	0.70	5.54	22	25	35	9.26	0.38	
12/1/2014	19	10.2	0.70	2.65	6	10		9.46	0.35	
12/8/2014	12	7.8	0.70	9.07	35	35		9.53	0.40	
12/15/2014	12	6.4	0.65	4.31	10	10		9.53	0.35	
12/22/2014	9	8.6	0.65	4.13	13	15		9.46	0.36	
			0.70	4.50	15.62	18.10	20.37		0.34	Average

Species: Murrah buffalo   Buffalo NO.: 2

Date(m/d/y)	Room temperature	Original semen			Diluted			equilibrium time	Motility after frozen
		Quantity (ml)	Sperm Motility	Density(10^9/ml)	Quantity of dilution buffer	Actual volume of addition	Total volume		
1/2/2014	20	7.1	0.76	7.71	26	30	30.76	9:55	0.32
1/6/2014	19	6.9	0.75	6.14	19	20	20.75	10:13	0.31
1/9/2014	13	6	0.77	10.68	32	35	35.77	10:07	0.34
1/13/2014	10	5.5	0.76	13.87	40	42	42.76	10:15	0.33
1/16/2014	8	6.4	0.74	10.71	35	35	35.74	9:48	0.33
1/20/2014	9	7.7	0.7	10.7	42	32	32.7	10:12	0.35
1/23/2014	6	6	0.75	5.87	15	15	15.75	9:54	0.39
1/27/2014	18	5.8	0.74	9.89	29	30	30.74	9:47	0.36
2/10/2014	9	3.5	0.74	7.27	12	15	15.74	9:47	0.31
2/13/2014	6	6.6	0.76	9.5	31	35	35.76	10:14	0.34
2/17/2014	11	9.4	0.77	6	24	38	38.77	9:50	0.33
2/20/2014	4	7.6	0.75	4.46	13	18	18.75	10:07	0.35
2/24/2014	13	7.2	0.78	6.6	21	24	24.78	10:27	0.35
2/27/2014	16.2	4.8	0.78	8.37	19	25	25.78	9:15	0.35
3/3/2014	13	5	0.76	7.19	17	20	20.76	9:53	0.3
3/6/2014	14	4.7	0.74	7.45	16	20	20.74	9:36	0.34
3/10/2014	11	8.1	0.73	4	11	15	15.73	9:19	0.32
3/13/2014	16	4.9	0.75	12.43	32	35	35.75	9:09	0.33
3/17/2014	15	4.7	0.75	8.4	19	19	19.75	9:05	0.3
3/20/2014	22	5.8	0.76	7.03	19	20	20.76	9:58	0.35
3/24/2014	21	7.5	0.76	11.13	43	43	43.76	9:55	0.4
3/27/2014	21	6.8	0.75	10.5	36	36	36.75	9:46	0.36
3/31/2014	24	6.3	0.78	9.98	31	38	38.78	10:06	0.4
4/7/2014	21	6.8	0.75	12.9	46	45	45.75	10:15	0.37
4/10/2014	24	6.3	0.77	8.33	25	36	36.77	10:06	0.34
4/14/2014	25	7.3	0.77	12.37	47	40	40.77	9:54	0.33
4/17/2014	25	5.9	0.77	6.24	16	16	16.77	10:09	0.35
4/21/2014	27	6.7	0.75	10.72	36	35	35.75	10:01	0.33
4/24/2014	25	6	0.76	5.71	15	16	16.76	10:30	0.33
4/28/2014	26	6.5	0.75	9.18	29	29	29.75	10:00	0.35

5/5/2014	20	8.7	0.75	11.96	54	40	40.75	10:36	0.34
5/8/2014	22	6.9	0.76	5.49	15.8	20	20.76	10:21	0.33
5/12/2014	27	5.4	0.77	8.77	23	33	33.77	10:33	0.36
5/15/2014	27	4.1	0.76	8.69	17	17	17.76	9:40	0.33
5/19/2014	27	6.9	0.77	8.23	27	40	40.77	10:08	0.38
5/22/2014	29	4.7	0.77	8.86	20	33	33.77	10:01	0.4
5/26/2014	31	5.3	0.75	7.93	20	20	20.75	10:47	0.35
5/29/2014	30	7.1	0.76	7.85	26	28	28.76	10:50	0.4
6/5/2014	30	8.9	0.77	6.36	25	35	35.77	9:57	0.39
6/9/2014	27	5.1	0.76	10.9	28	36	36.76	10:40	0.45
6/12/2014	26	7.8	0.76	7.6	27	39	39.76	10:09	0.35
6/16/2014	30	4.1	0.78	6.85		20	20.78	10:45	0.35
6/19/2014	29	5.6	0.76	8.85	24	28	28.76	10:09	0.35
6/23/2014	30	5.7	0.76	7.94	22	20	20.76	10:44	0.35
6/26/2014	30	6.3	0.78	6.06	17	18	18.78	9:16	0.4
6/30/2014	30	5.9	0.76	10.1	30	34	34.76	9:53	0.35
7/3/2014	30	7	0.77	7.15	22	22	22.77	9:06	0.3
7/7/2014	30	6.4	0.76	4.37	10	15	15.76	9:36	0.3
7/10/2014	30	5.8	0.74	7.18	19	19	19.74	9:49	0.32
7/14/2014	30	7.3	0.75	8.30	29	30	30.75	9:50	0.45
7/17/2014	31	8.7	0.77	6.53	25	25	25.77	9:24	0.40
7/21/2014	28	5.5	0.75	10.76	30	30	30.75	10:07	0.38
7/24/2014	30	5.1	0.72	6.83	16	19	19.72	9:39	0.32
7/28/2014	30	7.5	0.77	9.13	34	34	34.77	10:17	0.30
7/31/2014	31	6.0	0.77	8.78	26	26	26.77	10:06	0.40
8/4/2014	31	9.3	0.78	8.25	37	40	49.3	10:10	0.32
8/7/2014	30	6.6	0.78	8.08	25	25	31.6	9:49	0.30
8/11/2014	31	11.4	0.75	10.10	58	40	51.4	10:13	0.34
8/14/2014	24	9.3	0.76	6.33	26	30	39.3	9:58	0.35
9/11/2014	29	9.8	0.79	8.50	40	40	50	9:41	0.36
9/15/2014	31	7.9	0.78	9.21	36	40	48	10:10	0.50
9/18/2014	29	6.7	0.77	8.85	29	29	36	9:44	0.40
9/22/2014	29	6.9	0.79	10.38	36	43	50	9:54	0.30
9/25/2014	28	8.1	0.73	7.15	27	29	37	9:28	0.40
9/29/2014	28	7.6	0.75	4.45	13	15	23	10:20	0.37
10/9/2014	22	6	0.78	11.82	37	40	40.78	10:24	0.32
10/13/2014	24	8.5	0.78	9.12	38	40	40.78	9:03	0.3
10/16/2014	22	10.8	0.75	7.93	41	44	44.75	9:03	0.35
10/20/2014	27	7.1	0.8	10.15	36	40	40.8		0.35
10/23/2014	23	7.8	0.78	8.27	31	35	35.78	9:34	0.35

10/27/2014	26	5.8	0.77	4.56	10	15	15.77	9:55	0.3	
10/30/2014	27	8.2	0.78	11.76	72	70	70.78	11:07	0.43	
11/3/2014	16	7.6	0.79	10.52	40	40	48	10:05	0.46	
11/6/2014	20	6.6	0.78	6.32	18	28	35	10：33	0.40	
11/10/2014	18	4.4	0.78	9.58	21	26	30	10.04	0.45	
11/13/2014	17	6.6	0.79	10.91	37	46	53	9.46	0.38	
11/17/2014	17	6.0	0.77	4.97	12	26	32	10:21	0.45	
11/20/2014	18	5.8	0.74	7.86	22	25	31	9.35	0.45	
11/24/2014	19	8.9	0.76	8.59	37	36	45	10.36	0.45	
11/27/2014	18	7.5	0.78	10.54	40	35	43	10.32	0.48	
12/1/2014	19	6.4	0.78	8.34	26	30		10.28	0.35	
12/4/2014	14	5.8	0.77	7.72	21	23		9.40	0.46	
12/8/2014	12	7.1	0.76	9.85	35	35		10.22	0.35	
12/11/2014	13	8.3	0.77	10.80	46	46		9.55	0.40	
12/15/2014	12	5.6	0.76	13.50	40	46		11.05	0.38	
12/18/2014	12	7.5	0.76	10.21	38	40		9.56	0.34	
12/22/2014	9	3.7	0.75	6.49	11	26		10.26	0.40	
12/25/2014	15	6.2	0.75	10.09	31	35		11.30	0.40	
			0.76	8.57	28.16	30.64	32.09		0.36	Average

Species: Murrah Buffalo
buffalo NO.: 3

Date(m/d/y）	Room temperature	Original semen			Diluted				Motility after frozen
		Quantity (ml)	Sperm Motility	Density(10^9/ml)	Quantity of dilution buffer	Actual volume of addition	Total volume	equilibrium time	
1/2/2014	20	4.9	0.73	8.8	21	25	25.7	9:07	0.30
1/6/2014	19	6.6	0.75	9.2	30	35	35.8	9:08	0.37
1/9/2014	13	6.1	0.77	13.3	43	45	45.8	9:14	0.34
1/13/2014	10	6.8	0.77	8.2	27	36	36.8	9:17	0.37

1/16/2014	8	4.6	0.75	15.7	32	35	35.8	9:07	0.33
1/20/2014	9	5.2	0.76	7.9	39	39	39.8	9:09	0.31
1/23/2014	6	6.1	0.76	10.1	31	33	33.8	9:17	0.35
1/27/2014	18	4.3	0.75	11.1	24	26	26.8	9:00	0.40
2/10/2014	9	5.6	0.72	10.1	28	30	30.7	9:16	0.33
2/13/2014	6	4.8	0.68	10.0	24	25	25.7	9:05	0.32
2/17/2014	11	5.3	0.70	10.4	28	36	36.7	9:10	0.31
2/20/2014	4	6.4	0.74	9.7	31	35	35.7	9:29	0.36
2/24/2014	13	6.6	0.75	10.4	35	44	44.8	9:18	0.38
2/27/2014	16	5.2	0.75	13.6	37	40	40.8	9:10	0.32
3/3/2014	13	5.8	0.76	10.3	30	35	35.8	9:07	0.35
3/6/2014	14	2.8	0.74	9.0	12	18	18.7	8:57	0.37
3/10/2014	11	5.0	0.74	10.2	26	32	32.7	8:40	0.34
3/13/2014	16	3.8	0.75	14.3	32	32	32.8	9:13	0.30
3/17/2014	15	5.3	0.67	13.4	38	25	25.7	9:12	0.30
3/20/2014	22	4.8	0.60	13.5	34	34	34.6	10:28	0.34
3/24/2014		6.8	0.68	13.7	49				0.34
3/27/2014	21	5.4	0.70	9.3	24	25	25.7	10:02	0.34
3/31/2014	24	6.5	0.74	10.9	36	36	36.7	9:24	0.33
4/7/2014	21	6.9	0.76	19.8	75	70	70.8	9:36	0.35
4/10/2014		4.6	0.76	9.2	21	27	27.8	9:24	0.40
4/14/2014	25	6.1	0.76	11.8	37	36	36.8	9:27	0.35
4/17/2014	25	6.2	0.78	10.0	31	32	32.8	9:24	0.33
4/21/2014	27	8.5	0.76	8.9	37	37	37.8	9:20	0.35
4/24/2014		5.9	0.70	4.8	11	11	11.7	9:19	0.33
4/28/2014	26	6.6	0.75	9.4	31	35	35.8	9:12	0.32
5/5/2014	20	7.4	0.75	19.2	78	70	70.8	9:29	0.34
5/8/2014	22	6.2	0.76	7.8	23	30	30.8	9:20	0.35
5/12/2014	27	6.1	0.77	9.9	30	45	45.8	8:54	0.35
5/15/2014	27	3.2	0.76	13.0	22	28	28.8	9:03	0.40
5/19/2014	27	7.4	0.76	15.7	63	55	55.8	9:03	0.33
5/22/2014	29	6.7	0.75	6.6	20	50	50.8	9:07	0.30
5/26/2014	31	5.3	0.78	9.8	26	42	42.8	9:05	0.42
5/29/2014	30	6.0	0.76	10.4	31	35	35.8	9:15	0.35
6/5/2014	30	8.0	0.77	13.7	58	58	58.8	9:01	0.40
6/9/2014	27	7.0	0.75	11.7	42	50	50.8	9:05	0.40
6/12/2014	26	6.4	0.76	8.8	27	31	31.8	8:53	0.38
6/16/2014	30	5.0	0.77	10.7	27	35	35.8	9:10	0.35
6/19/2014	29	4.6	0.77	10.2	23	29	29.8	8:47	0.45
6/23/2014	30	4.8	0.77	12.0	30	32	32.8	9:01	0.35

6/26/2014	30	5.7	0.77	11.4	33	35	35.8	9:00	0.33
6/30/2014	30	4.6	0.76	9.9	23	25	25.8	10:58	0.38
7/3/2014	30	5.1	0.76	8.9	22	22	22.8	9:26	0.30
7/7/2014	30	5.6	0.76	14.6	44	58	58.8	9:46	0.35
7/10/2014	30	4.5	0.75	9.4	21	30	30.8	9:31	0.35
7/14/2014	30	7.0	0.76	9.6	34	33	33.8	10:44	0.33
7/17/2014	31	5.1	0.77	8.0	19	20	20.8	9:39	0.30
7/21/2014	28	7.7	0.75	3.8	10	10	10.8	10:13	0.38
7/24/2014	30	5.9	0.78	8.7	25	25	25.8	9:54	0.33
7/28/2014	30	5.4	0.76	11.1	31	31	31.8	10:27	0.37
7/31/2014	31	5.9	0.77	14.0	44	45	45.8	9:54	0.35
8/4/2014	31	6.5	0.80	11.13	37	40	46.5	10:40	0.34
8/7/2014	30	9.0	0.77	7.61	32	36	45.0	10:14	0.32
8/11/2014	31	5.3	0.77	6.92	17	15	20.3	10:30	0.33
8/14/2014	24	6.1	0.77	11.22	35	35	41.1	11:08	0.35
9/11/2014	29	7.7	0.72	12.00	47	50	58	9:55	0.40
9/15/2014	31	8.5	0.77	10.08	43	43	52	10:21	0.35
9/18/2014	29	5.9	0.78	6.78	18	18	24	9:55	0.35
9/22/2014	29	5.5	0.79	12.26	34	45	51	10:38	0.35
9/25/2014	28	2.0	0.76	16.12	17	17	19	11:25	0.35
9/29/2014	28	2.6	0.76	6.73	8	10	13	10:35	0.30
10/9/2014	22	5.2	0.75	13.8	34	35	35.8	11:00	0.32
10/16/2014	22	5.3	0.77	10.7	29	30	30.8	9:38	0.49
10/20/2014	27	4.6	0.79	9.5	22	30	30.8	10:29	0.38
10/23/2014	23	4.7	0.70	16.0	40	40	40.7	9:46	0.40
10/27/2014	26	6.0	0.78	11.9	37	40	40.8	10:20	0.43
11/3/2014	16	5.9	0.75	15.69	50	70	76	10:15	0.40
11/6/2014	20	5.8	0.78	11.92	36	50	56	10：20	0.43
11/10/2014	18	7.4	0.76	14.21	56	58	65	11.41	0.38
11/13/2014	17	6.2	0.77	9.02	27	30	36	9.55	0.35
11/17/2014	17	6.8	0.76	8.73	29	40	47	10:33	0.35
11/20/2014	18	4.9	0.78	10.08	25	32	37	10.26	0.34
11/24/2014	19	5.9	0.77	15.47	49	50	56	10.23	0.40
11/27/2014	18	6.0	0.77	13.93	44	44	50	10.02	0.45
12/1/2014	19	4.4	0.76	10.67	24	30		10.27	0.40
12/4/2014	14	4.9	0.74	13.78	36	40		10.04	0.47
12/8/2014	12	8.5	0.76	11.63	51	55		10.40	0.37
12/11/2014	13	7.9	0.75	15.42	65	60		9.37	0.35
12/15/2014	12	4.4	0.75	16.06	38	45		10.36	0.37
12/18/2014	12	9.2	0.76	9.25	42	46		9.58	0.38

12/22/2014	9	7.0	0.74	9.67	34	38		9.58	0.38	
12/25/2014	15	7.7	0.72	11.68	46	50		10.10	0.33	
			0.75	11.11	33.28	36.65	36.29		0.36	Average

Species: Murrah buffalo Buffalo NO.: 4

Date(m/d/y)	Room temperature	Original semen			Diluted				Motility after frozen
		Quantity (ml)	Sperm Motility	Density(10^9/ml)	Quantity of dilution buffer	Actual volume of addition	Total volume	equilibrium time	
1/2/2014	20	7.6	0.73	5.84	17	25	25.7	10:43	0.33
1/6/2014	19	4.9	0.75	6.57	14	16	16.8	10:43	0.35
1/9/2014	13	6.4	0.78	10.16	33	42	42.8	10:59	0.43
1/13/2014	10	5.0	0.70	6.09	13	15	15.7	11:08	0.33
1/16/2014	8	7.9	0.76	10.16	40	46	46.8	10:39	0.33
1/20/2014	9	4.9	0.70	11.66	29	29	29.7	10:55	0.33
1/23/2014	6	6.0	0.77	9.94	30	30	30.8	10:34	0.36
1/27/2014	18	3.2	0.75	10.41	17	19	19.8	11:10	0.38
2/13/2014	6	5.1	0.68	15.18	41	49	49.7	10:41	0.33
2/27/2014	16	3.1	0.76	19.58	33	35	35.8	10:41	0.32
3/3/2014	13	5.9	0.75	6.47	17	30	30.8	10:27	0.30
3/6/2014	14	3.1	0.74	7.76	11	15	15.7	10:36	0.36
3/10/2014	11	4.4	0.73	8.27	17	20	20.7	10:20	0.35
3/13/2014	16	5.1	0.70	13.82	37	35	35.7	10:11	0.32
3/17/2014	15	6.2	0.74	8.86	27	20	20.7	10:22	0.30
4/10/2014		6.7	0.76	10.07	33	36	36.8	10:44	0.40
4/14/2014	25	6.0	0.75	17.77	58	48	48.8	10:58	0.35
4/17/2014	25	13.3	0.75	9.47	62	50	50.8	10:33	0.33
4/21/2014	27	5.8	0.75	9.33	27	27	27.8	10:51	0.34
4/28/2014	26	8.3	0.70	9.22	38	38	38.7	10:27	0.32
5/5/2014	20	9.4	0.75	13.05	64	60	60.8	10:29	0.34
5/8/2014	22	8.4	0.76	5.00	17	22	22.8	10:34	0.30
5/12/2014	27	11.1	0.77	9.61	53	53	53.8	10:20	0.30
7/14/2014	30	9.9	0.76	13.31	69	72	72.8	11:06	0.40

7/17/2014	31	1.8	0.76	6.78	24	23	23.8	10:26	0.30	
7/21/2014	28	8.5	0.75	8.46	35	35	35.8	10:25	0.35	
7/24/2014	30	7.3	0.77	9.70	35	35	35.8	10:30	0.33	
7/28/2014	30	8.3	0.78	7.10	0	0	0.8	10:53	0.00	
7/31/2014	31	6.4	0.77	8.88	28	35	35.8	10:38	0.35	
8/4/2014	31	9.4	0.76	14.03	70	65	74.4	11:20	0.35	
8/7/2014	30	7.8	0.75	8.45	32	32	39.8	10:59	0.30	
8/11/2014	31	9.2	0.74	8.81	40	40	49.2	11:16	0.33	
8/14/2014	24	5.0	0.77	9.65	24	29	34.0	10:55	0.35	
9/11/2014	29	7.2	0.77	8.80	28	31	38	10:25	0.35	
9/15/2014	31	6.1	0.76	7.15	20	25	31	10:15	0.34	
9/18/2014	29	6.6	0.78	5.91	9	25	32	10:45	0.40	
9/22/2014	29	8.0	0.78	9.26	36	46	54	10:50	0.35	
9/25/2014	28	10.4	0.80	7.57	37	37	47	10:41	0.39	
9/29/2014	28	11.6	0.76	5.17	24	25	37	11:00	0.36	
10/9/2014	22	9.8	0.79	11.61	59	59	59.8	10:58	0.30	
10/13/2014	24	5.0	0.76	9.84	25	25	25.8	10:57	0.30	
10/16/2014	22	13.7	0.74	6.20	17	22	22.7	10:43	0.30	
10/20/2014	27	2.4	0.76	7.70	9	15	15.8		0.48	
10/23/2014	23	3.3	0.78	17.46	31	35	35.8	10:45	0.38	
10/30/2014	27	9.1	0.75	9.27	42	48	48.8	10:44	0.45	
11/3/2014	16	8.1	0.75	10.13	41	45	53	11:18	0.49	
11/6/2014	21	5.8	0.79	10.08	29	35	41	11:11	0.45	
11/10/2014	18	10.0	0.80	7.51	35	42	52	11.30	0.46	
11/13/2014	17	2.3	0.77	6.36	7	13	15	10.35	0.35	
11/20/2014	18	3.0	0.75	15.88	26	32	35	10.50	0.48	
11/24/2014	19	6.8	0.78	12.40	44	35	42	10.36	0.40	
12/4/2014	14	5.3	0.70	8.85	23	25		10.24	0.49	
12/8/2014	12	6.4	0.75	19.19	67	65		11.00	0.35	
			0.75	9.92	31.96	34.17	35.18		0.35	Average

Species: Murrah buffalo   Buffalo NO.: 5

Date(m/d/y)	Room temperature	Original semen			Diluted				Motility after frozen
		Quantity (ml)	Sperm Motility	Density(10^9/ml)	Quantity of dilution buffer	Actual volume of addition	Total volume	equilibrium time	
1/2/2014	20	9.7	0.77	5.54	23	30	30.8	8:57	0.32
1/6/2014	19	11.0	0.76	9.31	50	50	50.8	8:53	0.33
1/9/2014	13	8.9	0.76	6.61	26	31	31.8	8:57	0.32
1/13/2014	10	9.0	0.75	12.26	57	67	67.8	9:13	0.33
1/16/2014	8	9.2	0.76	12.90	62	50	50.8	8:49	0.33
1/20/2014	9	7.2	0.76	9.35	33	33	33.8	8:49	0.33
1/23/2014	6	8.8	0.76	4.12	13	23	23.8	8:50	0.37
1/27/2014	18	6.4	0.75	10.35	33	37	37.8	8:55	0.32
2/10/2014	9	7.6	0.73	7.24	25	29	29.7	9:03	0.33
2/13/2014	6	8.7	0.72	8.84	37	38	38.7	8:47	0.32
2/17/2014	11	10.5	0.76	9.11	47	50	50.8	9:07	0.30
2/20/2014	4	10.8	0.76	7.99	41	60	60.8	9:20	0.36
2/24/2014	13	7.9	0.76	12.72	52	58	58.8	9:11	0.31
2/27/2014	16	6.6	0.78	9.41	30	32	32.8	9:59	0.32
3/3/2014	13	11.1	0.77	8.05	43	50	50.8	10:23	0.33
3/6/2014	14	8.9	0.74	3.28	9	12	12.7	8:56	0.35
3/10/2014	11	10.9	0.71	7.17	36	40	40.7	9:51	0.32
3/13/2014	16	3.7	0.75	9.60	18	18	18.8	9:49	
3/17/2014	15	7.3	0.71	14.18	55	30	30.7	10:04	0.30
3/20/2014	22	11.0	0.76	6.53	32	32	32.8	10:25	0.33
3/24/2014		30.0	0.75	4.80	39				0.34
3/27/2014	21	7.3	0.73	12.70	49	48	48.7	10:10	0.36
3/31/2014	24	13.3	0.76	11.22	76	70	70.8	10:12	0.32
4/7/2014	21	18.7	0.76	15.04	150	78	78.8	10:23	0.33
4/10/2014		12.8	0.76	6.89	40	42	42.8	10:21	0.34
4/14/2014	25	8.2	0.73	13.55	59	60	60.7	10:14	0.30
4/17/2014	25	6.2	0.75	14.10	46	45	45.8	10:37	0.31
4/21/2014	27	13.3	0.76	7.13	44	44	44.8	10:21	0.34
4/24/2014		7.7	0.76	9.50	37	38	38.8	10:11	0.30

4/28/2014	26	12.8	0.75	8.22	50	50	50.8	10:11	0.30
5/5/2014	20	13.4	0.60	7.48	47	43	43.6	10:19	0.32
5/8/2014	22	12.7	0.75	9.26	58	60	60.8	10:28	0.31
5/12/2014	27	15.0	0.73	11.60	89	55	55.7	9:50	0.30
5/15/2014	27	13.4	0.75	16.07	116	90	90.8	10:01	0.32
5/19/2014	27	9.0	0.75	9.75	44	44	44.8	9:36	0.33
5/22/2014	29	16.3	0.76	5.10	34	38	38.8	10:07	0.32
5/26/2014	31	7.6	0.70	18.55	77	70	70.7	10:15	0.30
5/29/2014	30	10.0	0.76	7.97	38	38	38.8	10:22	0.32
6/5/2014	30	13.5	0.76	8.94	59	62	62.8	9:37	0.33
6/9/2014	27	10.1	0.75	8.87	44	50	50.8	8:27	0.31
6/12/2014	26	7.5	0.76	9.81	37	37	37.8	8:34	0.32
6/16/2014	30	11.4	0.76	7.37	39	38	38.8	8:33	0.33
6/19/2014	29	11.0	0.75	11.74	67	66	66.8	8:29	0.35
6/23/2014	30	12.7	0.75	7.88	47	50	50.8	8:35	0.30
6/26/2014	30	12.5	0.77	11.54	74	70	70.8	8:27	0.34
6/30/2014	30	11.8	0.77	9.20	53	58	58.8	8:51	0.30
7/3/2014	30	9.1	0.77	7.25	30	30	30.8	8:33	0.30
7/7/2014	30	11.2	0.75	3.63	25	33	33.8	8:35	0.30
7/10/2014	30	8.8	0.75	5.95	23	20	20.8	8:20	0.32
7/14/2014	30	12.6	0.75	10.86	70	70	70.8	8:29	0.30
7/17/2014	31	10.2	0.77	7.45	35	35	35.8	8:16	0.30
7/21/2014	28	10.6	0.73	3.55	12	12	12.7	8:47	0.10
7/24/2014	30	11.8	0.74	9.62	56	56	56.7	8:48	0.30
7/28/2014	30	11.7	0.74	7.40	40	39	39.7	8:40	0.30
7/31/2014	31	14.0	0.76	9.30	64	59	59.8	8:52	0.34
8/4/2014	31	7.9	0.65	6.57	23	25	32.9	8:26	0.31
8/7/2014	30	8.6	0.75	10.55	46	45	53.6	8:32	0.30
8/11/2014	31	11.5	0.71	11.71	69	60	71.5	8:31	0.30
8/14/2014	24	8.9	0.75	6.86	28	26	34.9	8:45	0.30
9/11/2014	29	8.5	0.77	7.80	31	30	39	8:35	0.30
9/15/2014	31	12.6	0.78	8.68	53	58	71	8:55	0.35
9/18/2014	29	8.2	0.77	12.69	54	55	63	8:39	0.35
9/22/2014	29	6.8	0.79	8.42	28	33	40	8:59	0.30
9/25/2014	28	10.6	0.78	8.09	41	60	71	8:50	0.43
9/29/2014	28	10.9	0.76	10.30	57	60	71	8:37	0.40
10/9/2014	22	8.1	0.78	9.14	36	52	52.8	8:30	0.30
10/13/2014	24	6.7	0.77	13.92	49	58	58.8	8:36	0.30
10/16/2014	22	6.0	0.75	12.17	38	40	40.8	8:48	0.45
10/20/2014	27	11.0	0.76	9.91	54	62	62.8	8:56	0.35

10/23/2014	23	3.9	0.77	14.77	31	37	37.8	8:48	0.35	
10/27/2014	26	14.5	0.76	7.30	56	62	62.8	8:55	0.44	
10/30/2014	27	7.8	0.74	12.99	53	53	53.7	8:54	0.45	
11/3/2014	16	7.0	0.78	11.34	41	45	52	8:52	0.47	
11/6/2014	20	6.2	0.75	9.40	29	36	42	9：01	0.45	
11/10/2014	18	7.4	0.76	11.22	42	45	52	8.58	0.48	
11/13/2014	17	6.9	0.74	10.79	38	45	52	9.05	0.37	
11/17/2014	17	9.8	0.73	4.84	19	28	38	9:03	0.36	
11/20/2014	18	6.3	0.78	7.52	22	32	38	9.18	0.45	
11/24/2014	19	8.0	0.77	18.79	82	64	72	9.12	0.35	
11/27/2014	18	6.7	0.76	13.48	48	50	57	8.55	0.34	
12/1/2014	19	8.7	0.75	13.67	63	70		8.52	0.40	
12/4/2014	14	6.7	0.75	14.90	53	58		9.08	0.32	
12/8/2014	12	8.7	0.77	10.54	46	50		9.04	0.40	
12/11/2014	13	15.0	0.76	13.04	102	80		9.39	0.38	
12/15/2014	12	12.6	0.75	10.82	69	69		9.27	0.34	
12/18/2014	12	12.5	0.75	10.60	67	67		9.17	0.34	
12/22/2014	9	8.1	0.73	15.58	68	70		9.22	0.39	
12/25/2014	15	14.5	0.77	5.83	36	46		9.20	0.34	
			0.75	9.75	47.29	47.57	47.25		0.34	Average

Species: Murrah buffalo   Buffalo NO.: 6

Date(m/d/y）	Room temperature	Original semen			Diluted				Motility after frozen
		Quantity (ml)	Sperm Motility	Density(10^9/ml)	Quantity of dilution buffer	Actual volume of addition	Total volume	equilibrium time	
1/8/2014	15	6.3	0.78	8.93	28	30	30.78	9:25	0.4
1/15/2014	6	2	0.65	14.96	16	18	18.65	9:35	0.34

1/22/2014	6	2.9	0.77	10.54	15	22	22.77	10:43	0.35	
2/8/2014	12	3.1	0.77	12.43	20	25	25.77	9:39	0.37	
4/7/2014	21	3.4	0.76	12.94	23	28	28.76	11:09	0.39	
4/10/2014		4.3	0.78	9.9	21	25	25.78	11:01	0.35	
4/14/2014	25	2.7	0.76	15.24	22	22	22.76	10:57	0.35	
4/23/2014	23	3.4	0.7	8.21	13	23	23.7	10:37	0.32	
5/7/2014	21	2.4	0.76	10.14	12	16	16.76	11:07	0.33	
5/11/2014	27	3.8	0.75	7.11	12	13	13.75	10:36	0.45	
5/17/2014	26	7.5	0.75	9.19	34	40	48	11:06	0.34	
5/24/2014	25	5.7	0.68	6.03	18	20	26	11:30	0.30	
10/8/2014	24	5.2	0.8	11.98	32.00	32	33	0	0.3	
10/13/2014	24	5	0.75	10.29	26	35	36	9:20	0.3	
10/20/2014	27	2.5	0.76	9.14	11	15		10:05	0.35	
10/29/2014	25	5.3	0.75	12.43	34	35	36	10:21	0.37	
11/3/2014	16	6.5	0.74	10.59	35	45	46	10:53	0.43	
11/10/2014	18	4.8	0.75	9.57	23	30	35	11.08	0.35	
11/17/2014	17	4.2	0.70	6.71	13	28	32	11:00	0.45	
11/24/2014	19	7.3	0.78	11.36	43	42	49	11.35	0.35	
12/1/2014	19	5.8	0.79	5.29	13	23		11.00	0.40	
12/8/2014	12	5.7	0.74	15.00	46	46		11.12	0.45	
12/16/2014	15	5.5	0.75	16.95	50	50		11.29	0.37	
12/22/2014	9	5.3	0.75	11.12	30	35		11.05	0.40	
			0.75	10.65	24.08	28.56	25.47	0.43	0.37	Average

Species: Murrah buffalo   Buffalo NO.: 7

Date(m/d/y)	Room temperature	Original semen			Diluted				Motility after frozen
		Quantity (ml)	Sperm Motility	Density(10^9/ml)	Quantity of dilution buffer	Actual volume of addition	Total volume	equilibrium time	
1/8/2014	15	10.3	0.77	11.63	62	65	65.77	9:50	0.35

1/15/2014	6	9	0.77	11.77	56	58	58.77	10:00	0.38
1/22/2014	6	9.9	0.77	13.52	70	70	70.77	6:14	0.36
2/8/2014	12	7.4	0.75	15.04	59	60	60.75	10:06	0.38
2/10/2014	11	4.3	0.74	13.97	32	35	35.74	11:10	0.4
2/12/2014	7	9.6	0.75	12.74	64	60	60.75	10:35	0.36
2/13/2014	16	11.2	0.66	17.47	105	95	95.66	10:40	0.31
2/17/2014	15	6.5	0.76	11.56	39	35	35.76	10:50	0.33
2/20/2014	22	9.2	0.7	11	52	50	50.7	11:09	0.35
2/24/2014		16	0.75	5.33	35		0.75		0.4
2/26/2014	16	12.8	0.76	11.43	75	80	80.76	9:49	0.33
2/27/2014	21	8.3	0.7	14.29	62	55	55.7	11:17	0.45
3/10/2014	11	4.3	0.74	13.97	32	35		11:10	0.4
3/13/2014	16	11.2	0.66	17.47	105	95		10:40	0.31
3/17/2014	15	6.5	0.76	11.56	39	35		10:40	0.33
3/20/2014	20	9.2	0.7	11	52	50		11:09	0.35
3/24/2014	20	16.0	0.75	5.33	35	40		11:09	0.4
3/27/2014	21	8.3	0.7	14.29	62	55		11:09	0.45
3/31/2014	24	7.9	0.75	10.8	43	45		11:09	0.33
4/7/2014	21	8	0.76	19.27	85	67	67.76	11:17	0.39
4/10/2014		9.5	0.78	9.7	46	50	50.78	11:10	0.38
4/14/2014	25	9.7	0.7	10.32	50	50	50.7	11:10	0.33
4/17/2014	25	7.9	0.7	14.48	61	56	56.7	11:02	0.34
4/21/2014	27	12	0.75	8.68	50	50	50.75	11:13	0.3
4/24/2014		6.5	0.7	9.51	31	30	30.7	10:40	0.3
4/28/2014	26	12.2	0.75	7.64	44	43	43.75	10:46	0.35
5/5/2014	20	12.7	0.7	9.52	60	50	50.7	10:46	0.3
5/8/2014	22	8	0.7	12.26	50.8	51	51.7	10:43	0.4
5/12/2014	27	10.3	0.75	12.17	65	62	62.75	10:55	0.35
5/15/2014	27	8.9	0.76	14.77	70	70	70.76	10:44	0.34
5/19/2014	27	8.4	0.78	14.88	67	63	63.78	11:10	0.33
5/22/2014	29	7.9	0.77	8.93	34	35	35.77	10:43	0.3
5/26/2014	31	8.4	0.75	9.68	40	45	45.75	11:14	0.35
5/29/2014	30	11	0.75	10.79	60	65	65.75	11:12	0.34
6/5/2014	30	14	0.76	6.79	43	60	60.76	10:47	0.33
6/9/2014	27	10.3	0.76	17.5	98	70	70.76	10:25	0.4
6/12/2014	26	8.9	0.7	10.79	49	49	49.7	10:59	0.37
6/16/2014	30	7.9	0.76	7.74		25	25.76	10:30	0.35
6/19/2014	29	9.8	0.76	10.64	53	55	55.76	10:45	0.34
6/23/2014	30	11.1	0.73	7.94	42	55	17:31	10:28	0.45
6/26/2014	30	12.8	0.76	8.24	50	50	18:14	10:19	0.31

6/27/2014	29	2.3	0.75	15.53	19	25	18:00	10:16	0.3
6/30/2014	30	12.4	0.75	8.74	53	45	18:00	10:49	0.45
7/3/2014	30	9.6	0.75	5.54	22	30	30.75	10:44	0.40
7/7/2014	30	11.8	0.76	7.32	40	40	40.76	11:51	0.30
7/10/2014	30	9.7	0.70	9.92	48	48	16:48	10:18	0.31
7/14/2014	30	9.5	0.76	10.25	49	45	18:14	10:45	0.39
7/17/2014	31	8.0	0.76	7.24	27	37	18:14	10:06	0.30
7/21/2014	28	10.4	0.72	5.75	26	35	17:16	10:47	0.34
7/24/2014	30	11.1	0.65	7.22	37	37	15:36	9:34	0.30
7/28/2014	30	9.5	0.70	7.27	32	32	16:48	9:15	0.30
7/31/2014	31	7.1	0.75	14.40	54	55	18:00	9:52	0.30
8/4/2014	31	11.0	0.75	9.43	52	49	60.0	9:36	0.35
8/7/2014	30	8.7	0.75	8.01	33	38	46.7	9:32	0.30
8/11/2014	31	7.0	0.72	6.32	19	19	26.0	9:32	0.30
8/14/2014	24	6.9	0.74	7.76	25	25	31.9	9:30	0.35
9/11/2014	29	10.4	0.78	8.00	40	40	50	10:28	0.30
9/15/2014	31	6.9	0.75	9.26	31	41	48	10:39	0.30
9/18/2014	29	9.1	0.70	11.20	52	52	61	9:40	0.34
9/22/2014	29	7.6	0.70	11.48	45	45	53	9:14	0.35
9/25/2014	28	6.6	0.73	7.14	22	22	29	9:53	0.35
9/29/2014	28	10.5	0.76	9.12	47	50	61	9:56	0.33
10/9/2014	22	11.7	0.75	13.1	80	70	70.75	9:18	0.3
10/13/2014	24	8.2	0.77	13.93	60	60		9:30	0.2
10/16/2014	22	8	0.7	8.51	33	32		9:42	0.3
10/20/2014	27	7.8	0.7	6.11	21	20		10:31	0.3
10/23/2014	23	10.6	0.75	10.56	57	56		9:50	0.4
10/27/2014	26	12.4	0.7	7.48	43	43		11:15	0.31
10/30/2014	27	7.3	0.75	9.71	35	40		10:12	0.35
11/1/2014	19	9.7	0.73	10.54	52	56		9.45	0.36
11/4/2014	14	5.7	0.75	8.92	25	30		10.03	0.37
11/6/2014	20	6.8	0.76	9.03	30	42	49	9：56	0.35
11/8/2014	12	7.2	0.72	16.00	62	60		10.15	0.38
11/10/2014	18	5.1	0.75	12.23	32	36	41	10.28	0.36
11/13/2014	17	6.9	0.76	8.11	37	40	47	9.50	0.38
11/15/2014	12	8.4	0.75	18.37	84	65		11.00	0.30
11/17/2014	17	7.1	0.70	6.56	21	20	27	10:06	0.32
11/18/2014	12	5.8	0.75	14.36	43	50		10.10	0.34
11/20/2014	18	6.0	0.76	11.97	37	40	46	10.09	0.45
11/22/2014	9	6.8	0.75	15.16	55	55		10.11	0.35
11/24/2014	19	6.0	0.70	10.31	31	30	36	10.05	0.32

11/25/2014	15	9.2	0.75	15.72	78	75		10.06	0.33	
11/27/2014	18	8.6	0.70	10.62	46	46	55	9.59	0.35	
			0.74	10.85	48.26	48.07	47.86		0.35	Average

Species: Murrah buffalo   Buffalo NO.: 8

Date(m/d/y)	Room temperature	Original semen			Diluted				Motility after frozen
		Quantity (ml)	Sperm Motility	Density(10^9/ml)	Quantity of dilution buffer	Actual volume of addition	Total volume	equilibrium time	
1/2/2014	20	8.9	0.77	8.01	24	27	27.8	9:34	0.32
1/6/2014	19	10.3	0.75	4.94	20	25	25.8	9:28	0.32
1/9/2014	13	7.2	0.75	12.17	45	50	50.8	9:52	0.35
1/16/2014	8	4.7	0.76	6.01	12	15	15.8	10:00	0.34
1/20/2014	9	6.5	0.78	12.84	44	50	50.8	9:59	0.33
1/23/2014	6	7.8	0.77	9.67	38	49	49.8	8:57	0.36
1/27/2014	18	5.7	0.77	10.69	31	36	36.8	9:57	0.32
2/10/2014	9	6.3	0.76	11.35	37	40	40.8	9:06	0.32
2/13/2014	6	6.0	0.75	9.43	28	28	28.8	9:38	0.34
2/17/2014	11	8.2	0.75	9.26	37	40	40.8	9:37	0.38
2/20/2014	4	6.2	0.75	10.61	33	43	43.8	9:54	0.34
2/24/2014	13	5.6	0.75	12.70	37	38	38.8	9:56	0.35
2/27/2014	16	7.1	0.76	11.49	42	42	42.8	9:35	0.31
3/3/2014	13	6.0	0.75	11.50	35	40	40.8	9:35	0.30
3/6/2014	14	5.4	0.76	11.70	33	37	37.8	9:06	0.36
3/10/2014	11	6.8	0.72	9.81	33	35	35.7	9:30	0.35
3/13/2014	16	7.3	0.76	9.20	33	43	43.8	9:22	0.32
3/17/2014	15	7.0	0.74	16.81	64	55	55.7	9:45	0.32
3/20/2014	22	8.1	0.76	11.05	46	45	45.8	9:37	0.32
3/24/2014		5.8	0.74	15.77	49				0.38
3/27/2014	21	8.4	0.73	10.82	46	40	40.7	10:20	0.31
3/31/2014	24	6.8	0.76	12.28	43	45	45.8	9:41	0.33
4/7/2014	21	8.6	0.72	14.61	67	67	67.7	9:57	0.33
4/10/2014		6.6	0.78	11.83	40	40	40.8	9:44	0.35
4/14/2014	25	7.7	0.74	13.33	54	44	44.7	9:44	0.30
4/17/2014	25	8.0	0.77	7.15	26	26	26.8	9:42	0.35

4/21/2014	27	8.2	0.76	11.41	48	45	45.8	9:41	0.35
4/24/2014		7.3	0.77	5.80	19	20	20.8		0.33
4/28/2014	26	6.6	0.76	8.22	26	31	31.8	9:38	0.40
5/5/2014	20	7.6	0.70	14.45	58	50	50.7	9:49	0.33
5/8/2014	22	8.1	0.77	6.79	25	33	33.8	9:51	0.30
5/12/2014	27	6.4	0.77	18.50	64	59	59.8	9:24	0.33
5/15/2014	27	6.9	0.75	13.21	48	45	45.8	9:29	0.33
5/19/2014	27	5.8	0.76	19.33	62	60	60.8	9:30	0.30
5/22/2014	29	5.9	0.68	17.00	54	55	55.7	9:38	0.33
5/26/2014	31	6.6	0.70	13.00	45	55	55.7	9:30	0.30
5/29/2014	30	9.5	0.76	9.78	46	45	45.8	9:41	0.34
6/5/2014	30	8.8	0.76	11.75	53	50	50.8	9:32	0.36
6/9/2014	27	5.9	0.70	9.60	11	30	30.7	9:53	0.40
6/12/2014	26	5.9	0.65	9.54	28	28	28.7	9:39	0.30
6/16/2014	30	9.7	0.67	15.20	79	79	79.7	9:44	0.30
6/19/2014	29	5.2	0.77	8.95	23	28	28.8	9:19	0.30
6/23/2014	30	5.9	0.77	11.96	36	36	36.8	9:38	0.31
6/26/2014	30	4.5	0.78	7.73	16	20	20.8	9:35	0.32
6/30/2014	30	8.1	0.76	7.63	29	30	30.8	10:04	0.34
7/3/2014	30	4.9	0.77	10.42	26	30	30.8	9:54	0.33
7/7/2014	30	8.0	0.75	10.23	42	42	42.8	9:10	0.30
7/10/2014	30	8.4	0.77	8.90	37	40	40.8	9:00	0.34
7/14/2014	30	6.7	0.70	12.53	44	40	40.7	9:20	0.33
7/17/2014	31	8.6	0.75	9.40	40	45	45.8	9:11	0.30
7/24/2014	30	9.5	0.78	7.32	33	33	33.8	9:03	0.32
7/28/2014	30	6.8	0.76	9.29	32	32	32.8	9:01	0.33
8/4/2014	31	9.4	0.73	16.49	84	70	79.4	9:10	0.38
8/7/2014	30	5.6	0.78	8.03	21	35	40.6	9:09	0.35
8/11/2014	31	7.3	0.78	14.00	54	54	61.3	8:59	0.35
8/14/2014	24	6.1	0.77	5.69	15	15	21.1	9:04	0.35
9/11/2014	29	6.4	0.77	15.00	51	51	57	8:50	0.45
9/15/2014	31	9.9	0.76	11.00	55	58	68	9:11	0.33
9/18/2014	29	8.7	0.77	8.07	33	33	42	9:00	0.55
9/22/2014	29	7.6	0.78	10.75	41	50	58	9:06	0.30
9/25/2014	28	5.3	0.78	4.78	10	20	25	9:00	0.40
9/29/2014	28	8.4	0.75	9.66	40	43	51	9:11	0.35
10/9/2014	22	8.9	0.8	9.86	44.00	44		9:15	0.3
10/13/2014	24	9.1	0.77	10.37	48	51		9:06	0.30
10/16/2014	22	7.4	0.78	10.76	40	40		8:47	0.40
10/20/2014	27	6.6	0.78	13.57	47	55		9：16	0.38

10/23/2014	23	7.8	0.76	13.13	54	54		8：57	0.42	
10/27/2014	26	9.4	0.80	9.03	42	45		9：35	0.45	
10/30/2014	27	6.1	0.78	9.53	29	32		8：59	0.39	
11/3/2014	16	9.1	0.76	11.65	55	55	64	9:24	0.42	
11/6/2014	20	7.8	0.77	13.63	56	60	68	9：18	0.35	
11/10/2014	18	7.0	0.78	12.11	44	50	57	9.50	0.36	
11/13/2014	17	9.6	0.76	9.98	48	52	62	9.12	0.38	
11/17/2014	17	10.5	0.75	6.15	28	35	46	9:31	0.30	
11/20/2014	18	6.0	0.72	13.29	42	42	48	9.25	0.38	
11/24/2014	19	7.2	0.78	15.14	58	60	67	9.14	0.40	
11/27/2014	18	5.5	0.75	13.33	39	38	44	9.20	0.35	
12/1/2014	19	8.0	0.78	12.29	51	56		9.33	0.35	
12/4/2014	14	8.3	0.75	9.41	39	45		9.20	0.40	
12/8/2014	12	6.0	0.74	10.98	34	40		9.40	0.35	
12/11/2014	13	3.4	0.75	14.99	27	30		9.09	0.33	
12/15/2014	12	8.1	0.75	11.57	48	65		9.40	0.35	
12/18/2014	12	6.8	0.76	12.20	43	45		9.19	0.40	
12/22/2014	9	6.4	0.69	15.33	53	54		9.23	0.35	
12/25/2014	15	5.4	0.76	11.01	30	35		9.23	0.35	
			0.75	11.13	40.28	42.54	41.88		0.35	Average

Species: Murrah buffalo   Buffalo NO.: 9

Date(m/d/y)	Room temperature	Original semen			Diluted				Motility after frozen
		Quantity (ml)	Sperm Motility	Density(10^9/ml)	Quantity of dilution buffer	Actual volume of addition	Total volume	equilibrium time	
1/2/2014	20	8.9	0.75	4.64	16	20	20.75	10:29	0.32
1/6/2014	19	7.6	0.77	5.68	18	20	20.77	9:51	0.32
1/9/2014	13	8.9	0.76	5.6	21	26	26.76	9:41	0.4
1/13/2014	10	6.1	0.75	8.22	24	24	24.75	9:49	0.35

1/16/2014	8	7.2	0.78	14.17	54	50	50.78	9:45	0.35
1/20/2014	9	8.2	0.7	12.16	52	45	45.7	10:21	0.3
1/23/2014	6	5	0.7	3.23	5	6	6.7	10:07	0.3
1/27/2014	18	9.3	0.76	5.94	24	25	25.76	11:19	0.32
2/10/2014	9	15.2	0.75	7.17	50	50	50.75	9:57	0.32
2/13/2014	6	6.3	0.74	5.96	16	16	16.74	9:53	0.32
2/17/2014	11	9.3	0.76	8.83	40	41	41.76	9:58	0.3
2/20/2014	4	4.9	0.76	5.31	11	17	17.76	10:22	0.34
2/24/2014	13	3.9	0.75	6.87	12	16	16.75	10:50	0.36
2/27/2014	16.2	9.7	0.77	3.9	13	18	18.77	10:05	0.3
3/3/2014	13	9	0.75	3.97	12	14	14.75	10:00	0.33
3/6/2014	14	6.5	0.74	6.41	19	25	25.74	9:40	0.35
3/10/2014	11	6.3	0.74	3.51	7	10	10.74	10:06	0.4
3/13/2014	16	11.9	0.7	6.96	38	37	37.7	9:51	0.3
3/17/2014	15	9.6	0.74	9.18	43	30	30.74	10:03	0.32
3/20/2014	22	9.7	0.7	6.66	29	32	32.7	10:18	0.32
3/24/2014		14	0.77	2.93	11		0.77	11:18	0.33
3/27/2014	21	8.4	0.75	15.4	69	25	25.75	9:16	0.33
3/31/2014	24	8.7	0.77	5.6	21	19	19.77	10:24	0.35
4/7/2014	21	11.7	0.68	10.47	62	56	56.68	10:28	0.32
4/10/2014		9.8	0.78	8.42	40	35	35.78	10;34	0.32
4/14/2014	25	8.1	0.76	7.16	27	37	37.76	10:07	0.33
4/17/2014	25	11.4	0.78	6.8	35	35	35.78	9:56	0.33
4/21/2014	27	10.6	0.75	5.85	27	27	27.75	9:49	0.3
4/24/2014		9.6	0.7	2.2	3	25	25.7	10:11	0.3
4/28/2014	26	10.8	0.75	2.22	4	20	20.75	9:50	0.32
5/5/2014	20	9.97	0.68	11.57	59	55	55.68	9:55	0.3
5/8/2014	22	9.2	0.77	6.38	26	30	30.77	9:59	0.35
5/12/2014	27	13.9	0.7	7.49	48.6	65	65.7	9:35	0.3
5/15/2014	27	7.8	0.76	5.83	20	20	20.76	9:43	0.35
5/19/2014	27	12.4	0.77	5.95	32	36	36.77	9:49	0.32
5/22/2014	29	12.6	0.77	5.66	30	37	37.77	10:03	0.3
5/26/2014	31	13.4	0.7	5.47	31	32	32.7	9:55	0.35
5/29/2014	30	10.6	0.76	8.2	42	41	41.76	9:52	0.3
6/5/2014	30	9.2	0.75	11.2	53	55	55.75	9:59	0.33
6/9/2014	27	12.2	0.75	11.38	71	70	70.75	10:26	0.33
6/12/2014	26	10.9	0.76	5.83	27	26	26.76	10:34	0.33
6/16/2014	30	10.8	0.78	6.92	26	26	26.78	10:29	0.3
6/19/2014	29	7.6	0.77	3.75	10	23	23.77	9:48	0.4
6/23/2014	30	12.3	0.76	6.09	33	33	33.76	10:13	0.34

6/26/2014	30	8	0.78	6.45	23	25	25.78	10:14	0.35	
6/30/2014	30	14.6	0.65	6.01	38	37	37.65	10:48	0.3	
7/3/2014	30	7.7	0.78	3.32	8	10	10.78	10:22	0.3	
7/7/2014	30	11	0.76	6.78	34	34	34.76	10:10	0.32	
7/10/2014	30	9.2	0.76	8.1	36	35	35.76	9:52	0.3	
7/14/2014	30	7.4	0.77	8.12	29	30	30.77	10:28	0.34	
7/17/2014	31	8.8	0.77	7.03	28	30	30.77	9:52	0.30	
7/21/2014	28	6.7	0.77	4.14	10	10	10.77	9:57	0.30	
7/24/2014	30	9.8	0.78	7.13	32	32	32.78	9:35	0.33	
7/28/2014	30	11.1	0.78	7.51	39	40	40.78	9:40	0.33	
7/31/2014	31	8.2	0.78	5.34	18	18	18.78	10:15	0.30	
8/4/2014	31	9.4	0.65	10.05	47	45	54.4	9:40	0.30	
8/7/2014	30	8.7	0.76	9.84	43	43	51.7	9:20	0.35	
8/11/2014	31	13.2	0.73	7.79	49	49	62.2	9:33	0.33	
8/14/2014	24	8.8	0.72	7.90	33	30	38.8	9:24	0.35	
9/11/2014	29	8.8	0.74	7.90	33	35	44	9:25	0.30	
9/15/2014	31	10.9	0.76	10.36	57	58	69	9:41	0.40	
9/22/2014	29	8.7	0.78	9.57	41	52	61	9:43	0.35	
9/25/2014	28	7.4	0.78	7.78	27	27	34	11:00	0.40	
10/9/2014	22	5.6	0.77	15.2	46	45	45.77	10:40	0.3	
10/13/2014	24	4.7	0.77	10.97	26	25	25.77	10:39	0.3	
10/16/2014	22	4.2	0.8	11.69	25	25	25.8	10:06	0.35	
10/23/2014	23	4.6	0.8	17.72	44	46	46.8	10:18	0.37	
10/27/2014	26	3.1	0.76	11.99	19	23	23.76	9:18	0.46	
10/30/2014	27	3.7	0.77	12.14	23	30	30.77	10:09	0.4	
11/3/2014	16	4.5	0.76	15.16	36	46	51	10:31	0.35	
11/10/2014	18	7.5	0.75	12.50	49	50	58	11.13	0.37	
11/13/2014	17	5.8	0.77	7.63	21	30	36	10.41	0.36	
11/17/2014	17	7.0	0.74	4.85	13	25	32	10:46	0.45	
11/20/2014	18	5.3	0.77	6.34	15	20	25	10.06	0.45	
11/24/2014	19	5.7	0.76	8.15	22	25	31	9.58	0.43	
11/27/2014	18	7.7	0.76	11.45	45	45	53	9.50	0.40	
12/1/2014	19	6.5	0.76	13.92	48	50		10.09	0.36	
12/4/2014	14	9.2	0.75	6.74	28	32		9.46	0.47	
12/11/2014	13	6.4	0.76	11.92	39	40		9.47	0.40	
12/18/2014	12	7.1	0.77	8.11	27	30		9.43	0.40	
12/22/2014	9	9.2	0.76	7.59	33	36		10.20	0.39	
12/25/2014	15	6.9	0.77	9.31	32	38		9.37	0.35	
			0.75	7.90	30.82	32.73	33.64		0.34	Average

Species: Murrah buffalo Buffalo NO.: 10

Date(m/d/y)	Room temperature	Original semen			Diluted				Motility after frozen
		Quantity (ml)	Sperm Motility	Density(10^9/ml)	Quantity of dilution buffer	Actual volume of addition	Total volume	equilibrium time	
1/2/2014	20	7.0	0.68	11.63	42	45	45.7	11:13	0.33
1/27/2014	18	5.9	0.72	10.51	31	31	31.7	11:09	0.33
2/10/2014	9	11.9	0.68	6.93	38	40	40.7	11:00	0.31
3/7/2014	14	6.5	0.75	13.93	45	50	50.8	8:40	0.36
3/31/2014	24	7.1	0.69	15.56	58	47	47.7	11:08	0.36
4/7/2014	21	6.4	0.74	14.17	48	50	50.7	11:10	0.42
4/10/2014		2.2	0.65	5.30	4	5	5.7	10:43	0.32
4/17/2014	25	5.0	0.76	12.33	32	32	32.8	10:54	0.35
4/21/2014	27	2.6	0.75	3.12	2	3	3.8	11:09	0.34
4/24/2014		3.2	0.75	10.75	17		0.8	11:24	0.34
4/28/2014	26	2.1	0.76	3.86	3	5	5.8	10:57	0.31
5/12/2014	27	2.1	0.75	9.63	10	11	11.8	10:22	0.33
5/15/2014	27	2.0	0.75	11.47	16	16	16.8	10:24	0.31
5/19/2014	27	2.1	0.70	6.24	6	8	8.7	10:45	0.33
5/26/2014	31	2.7	0.75	8.00	10	15	15.8	10:32	0.40
6/5/2014	30	4.6	0.70	14.86	36	36	36.7	11:05	0.34
6/9/2014	27	3.1	0.76	4.13	5	15	15.8	10:18	0.32
6/12/2014	26	2.5	0.70	9.80	12	10	10.7	9:54	0.30
6/16/2014	30	5.9	0.75	14.43		39	39.8	10:30	0.33
6/23/2014	30	7.6	0.69	7.91	29	28	28.7	10:20	0.50
6/26/2014	30	5.9	0.78	8.40	24	25	25.8	10:05	0.45

6/30/2014	30	7.6	0.76	9.59	36	38	38.8	10:52	0.40	
7/3/2014	30	6.6	0.77	7.82	24	24	24.8	10:08	0.33	
7/7/2014	30	5.3	0.75	10.48	28	28	28.8	10:08	0.35	
7/10/2014	30	5.1	0.71	7.46	18	18	18.7	10:37	0.31	
7/14/2014	30	7.2	0.76	7.25	24	20	20.8	10:31	0.33	
7/17/2014	31	3.9	0.77	7.30	13	15	15.8	10:03	0.35	
7/21/2014	28	7.1	0.75	6.75	22	23	23.8	10:44	0.32	
7/24/2014	30	7.7	0.76	10.22	40	40	40.8	9:59	0.33	
7/28/2014	30	7.3	0.76	5.39	17	17	17.8	10:50	0.33	
7/31/2014	31	7.1	0.76	9.09	32	32	32.8	10:59	0.30	
8/4/2014	31	9.2	0.75	6.79	28	27	36.2	10:30	0.32	
8/7/2014	30	5.5	0.76	6.52	16	16	21.5	10:22	0.30	
8/11/2014	31	7.7	0.73	7.32	26	26	33.7	10:40	0.34	
9/11/2014	29	8.6	0.75	14.20	65	65	74	10:00	0.45	
9/15/2014	31	7.5	0.76	7.89	28	40	48	10:18	0.35	
9/18/2014	29	4.1	0.80	8.56	17	17	21	10:18	0.40	
9/22/2014	29	9.1	0.78	6.49	26	40	49	10:30	0.35	
9/25/2014	28	1.6	0.78	14.81	13	15	17	10:14	0.45	
10/9/2014	22	7.9	0.78	13.36	55	58	58.8	10:44	0.33	
10/13/2014	24	4.4	0.77	12.91	30	34	34.8	9:10	0.35	
10/16/2014	22	5.0	0.76	6.38	14	18	18.8	10:30	0.40	
10/20/2014	27	8.2	0.77	5.83	20	24	24.8	10:28	0.45	
10/23/2014	23	5.9	0.76	8.52	24	24	24.8	11:10	0.40	
10/27/2014	26	5.0	0.78	10.46	26	30	30.8	10:58	0.38	
10/30/2014	27	6.7	0.78	9.74	33	36	36.8		0.46	
11/3/2014	16	5.5	0.78	7.00	18	20	26	10:53	0.49	
11/17/2014	17	6.0	0.75	5.46	14	25	31	10:39	0.45	
11/20/2014	18	4.3	0.70	5.42	10	20	24	11.17	0.40	
11/24/2014	19	8.2	0.75	5.95	21	25	33	11.17	0.40	
11/27/2014	18	5.5	0.75	4.07	8	9	15	10.57	0.38	
12/1/2014	19	8.7	0.77	6.93	28	30		10.48	0.35	
12/4/2014	14	4.3	0.76	13.68	31	32		10.30	0.45	
12/8/2014	12	7.5	0.75	8.66	32	35		10.50	0.46	
12/11/2014	13	5.9	0.70	5.09	12	14		10.57	0.33	
			0.75	8.84	24.38	26.78	26.29		0.37	Average

2014 low motility sperm record

Species：Murrah buffalo   Buffalo NO.：1

date(m/d/y)		Original semen		
	Room temperature	Quantity (ml)	Motility	Density(10^9/ml)
1/2/2014	20	6.4	0.49	11.28
1/6/2014	19	4.4	0.55	2.65
1/9/2014	13	3.0	0.40	5.45
1/13/2014	10	6.0	0.55	5.61
1/16/2014	8	4.9	0.50	11.62
1/20/2014	9	4.3	0.48	15.46
1/23/2014	6	5.4	0.50	2.51
1/27/2014	18	5.2	0.52	12.01
2/10/2014	9	9.5	0.49	2.39
2/13/2014	6	5.5	0.47	7.25
2/17/2014	11	6.6	0.45	5.55
2/20/2014	4	5.0	0.30	5.15
2/24/2014	13	4.2	0.40	6.14
3/3/2014	13	5.4	0.40	12.64
3/6/2014	14	3.1	0.45	6.41
3/10/2014	11	6.8	0.40	6.90
3/13/2014	16	5.2	0.20	12.20
3/17/2014	15	6.8	0.30	5.34
3/20/2014	22	6.3	0.40	7.02
3/24/2014		5.4	0.55	3.35
3/27/2014	21	8.1	0.40	4.10
3/31/2014	24	5.3	0.20	5.64
4/7/2014	21	4.9	0.01	7.57
4/10/2014		5.6	0.00	4.21
4/17/2014	25	5.6	0.10	3.51
4/21/2014	27	4.8	0.10	2.47
4/24/2014		6.1	0.01	
4/28/2014	26	6.1	0.01	2.24
5/5/2014	20	5.0	0.01	2.91
5/8/2014	22	4.0	0.10	4.61
5/12/2014	27	5.9	0.50	1.51
5/19/2014	27	6.8	0.50	6.72
5/26/2014	31	5.2	0.45	7.85
6/5/2014	30	10.1	0.45	6.64
6/9/2014	27	6.7	0.50	5.19

6/16/2014	30	8.9	0.49	4.97	
6/23/2014	30	6.2	0.57	9.66	
6/30/2014	30	13.0	0.55	6.63	
7/7/2014	30	8.3	0.53	12.64	
7/14/2014	30	4.7	0.55	8.40	
7/21/2014	28	8.2	0.00	6.85	
7/28/2014	30	9.5	0.55	1.03	
8/4/2014	31	8.0	0.56	9.33	
8/11/2014	31	8.7	0.52	8.26	
8/14/2014	24	6.6	0.55	7.47	
9/11/2014	29	7.3	0.55	8.61	
9/15/2014	31	5.2	0.47	8.94	
9/22/2014	29	9.9	0.52	6.67	
9/29/2014	28	10.7	0.55	8.85	
10/9/2014	22	7.8	0.47	6.88	
10/16/2014	22	7.6	0.55	7.77	
10/20/2014	27	9.4	0.48	10.48	
10/27/2014	26	7.2	0.54	6.99	
11/3/2014	16	6.3	0.45	9.56	
11/10/2014	18	6.6	0.54	6.15	
11/17/2014	17	4.1	0.47	5.89	
11/24/2014	19	4.5	0.57	11.61	
12/1/2014	19	6.5	0.55	4.03	
12/8/2014	12	6.8	0.40	9.88	
12/15/2014	12	6.0	0.40	11.64	
12/22/2014	9	6.7	0.40	5.40	
			0.41	6.94	Average

Species：Murrah buffalo　　Buffalo NO.：2

date(m/d/y）	Room temperature	Original semen		
		Quantity (ml)	Motility	Density(10^9/ml)
1/2/2014	20	13.1	0.48	7.02
1/6/2014	19	8.7	0.53	10.81
1/9/2014	13	13.5	0.50	7.66
1/13/2014	10	11.4	0.50	10.43
1/16/2014	8	11.7	0.54	12.11
1/20/2014	9	10.4	0.45	15.01
1/23/2014	6	10.9	0.50	6.47

1/27/2014	18	5.9	0.40	5.25	
2/10/2014	9	7.4	0.30	10.69	
2/13/2014	6	6.7	0.30	11.59	
2/17/2014	11	6.9	0.10	8.78	
3/3/2014	13	5.6	0.40	10.00	
3/6/2014	14	7.1	0.40	6.66	
3/10/2014	11	9.3	0.40	6.80	
3/13/2014	16	10.2	0.30	8.60	
3/17/2014	15	9.0	0.48	7.12	
3/20/2014	22	5.1	0.40	5.38	
4/10/2014		12.3	0.47	15.21	
4/14/2014	25	7.8	0.31	7.43	
4/17/2014	25	9.3	0.45	9.16	
5/5/2014	20	10.5	0.45	8.89	
5/8/2014	22	12.9	0.47	8.86	
5/22/2014	29	7.5	0.55	8.31	
5/26/2014	31	11.6	0.47	6.97	
5/29/2014	30	5.0	0.56	3.97	
6/5/2014	30	17.8	0.30	8.55	
6/12/2014	26	4.4	0.45	13.22	
6/16/2014	30	6.9	0.30	13.90	
6/19/2014	29	9.7	0.53	7.75	
6/23/2014	30	3.1	0.50	13.19	
7/7/2014	30	9.0	0.47	9.71	
7/21/2014	28	3.6	0.45	9.61	
7/28/2014	30	9.8	0.55	9.61	
8/11/2014	31	9.9	0.52	6.44	
9/18/2014	29	5.5	0.56	9.20	
9/22/2014	29	7.4	0.57	1.35	
9/25/2014	28	7.7	0.58	7.25	
9/29/2014	28	2.6	0.57	8.52	
10/16/2014	22	8.5	0.58	7.84	
10/30/2014	27	11.9	0.51	8.48	
11/13/2014	17	7.7	0.50	11.01	
12/4/2014	14	8.3	0.40	14.47	
12/11/2014	13	8.4	0.52	21.82	
12/15/2014	12	5.0	0.40	17.60	
12/22/2014	9	10.4	0.40	10.13	
			0.45	9.53	Average

Species： Murrah buffalo | Buffalo NO.： 3

date(m/d/y）		Original semen		
	Room temperature	Quantity (ml)	Motility	Density(10^9/ml)
1/2/2014	20	8.1	0.35	2.75
1/6/2014	19	12.2	0.36	2.54
1/9/2014	13	17.5	0.30	2.60
1/13/2014	10	12.8	0.30	3.68
1/16/2014	8	22.4	0.35	6.31
1/20/2014	9	13.0	0.30	8.52
1/23/2014	6	15.4	0.30	3.15
1/27/2014	18	12.6	0.36	4.06
2/10/2014	9	22.7	0.30	2.87
2/13/2014	6	17.6	0.31	3.05
2/17/2014	11	17.0	0.35	2.99
2/20/2014	4	11.2	0.35	3.34
2/24/2014	13	23.0	0.30	4.28
2/27/2014	16	16.7	0.30	3.26
3/3/2014	13	19.5	0.36	4.51
3/6/2014	14	13.8	0.32	3.07
3/10/2014	11	11.6	0.34	2.76
3/13/2014	16	20.4	0.30	3.73
3/17/2014	15	15.1	0.30	8.34
3/20/2014	22	9.5	0.35	2.69
3/24/2014		11.3	0.30	4.83
3/27/2014	21	10.2	0.30	4.71
3/31/2014	24	12.0	0.30	4.78
4/7/2014	21	14.1	0.30	8.80
4/10/2014		11.9	0.30	3.64
4/14/2014	25	19.5	0.35	5.69
4/17/2014	25	17.9	0.35	5.06
4/21/2014	27	16.4	0.35	5.01
4/24/2014		16.1	0.30	4.34
4/28/2014	26	17.1	0.30	3.89
5/5/2014	20	21.5	0.35	6.25
5/8/2014	22	17.0	0.35	4.31
5/12/2014	27	20.7	0.30	8.23
5/15/2014	27	17.1	0.30	5.85

5/19/2014	27	19.1	0.30	4.98
5/22/2014	29	19.2	0.36	2.84
5/26/2014	31	18.5	0.35	7.08
5/29/2014	30	16.8	0.35	3.78
5/30/2014	29	5.7	0.36	2.09
6/5/2014	30	11.8	0.30	4.93
6/9/2014	27	22.0	0.32	4.30
6/12/2014	26	20.4	0.35	3.44
6/16/2014	30	17.9	0.35	4.36
6/19/2014	29	17.7	0.36	3.75
6/23/2014	30	18.3	0.36	4.94
6/26/2014	30	16.9	0.35	4.10
6/30/2014	30	18.4	0.32	3.77
7/3/2014	30	19.7	0.32	4.10
7/7/2014	30	19.0	0.30	5.97
7/10/2014	30	16.3	0.35	4.47
7/14/2014	30	18.6	0.35	3.43
7/17/2014	31	18.7	0.35	2.92
7/21/2014	28	9.8	0.32	3.72
7/28/2014	30	15.1	0.30	4.12
7/31/2014	31	13.2	0.35	3.38
8/4/2014	31	12.4	0.35	4.33
8/7/2014	30	11.7	0.35	3.59
8/11/2014	31	11.9	0.36	4.64
8/14/2014	24	10.3	0.30	7.30
9/11/2014	29	17.6	0.38	4.50
9/15/2014	31	19.4	0.40	3.40
9/18/2014	29	15.5	0.44	3.26
9/22/2014	29	16.4	0.47	5.65
9/29/2014	28	18.7	0.36	4.02
10/9/2014	22	23.4	0.3	5.56
10/13/2014	24	10.5	0.36	4.49
10/16/2014	22	9.4	0.38	4.10
10/20/2014	27	17.4	0.35	6.23
10/23/2014	23	18.6	0.30	3.68
10/27/2014	26	18.1	0.30	2.71
10/30/2014	27	13.0	0.35	4.59
11/3/2014	16	13.6	0.35	3.55
11/6/2014	20	15.4	0.36	3.89
11/10/2014	18	6.1	0.36	15.90

11/13/2014	17	16.2	0.36	4.47	
11/17/2014	17	17.8	0.32	1.66	
11/20/2014	18	16.1	0.35	5.65	
11/24/2014	19	18.1	0.36	4.44	
11/27/2014	18	12.9	0.35	6.80	
12/1/2014	19	16.5	0.35	4.36	
12/4/2014	14	8.9	0.35	4.41	
12/8/2014	12	17.97	0.40	7.80	
12/11/2014	13	17.6	0.40	9.53	
12/15/2014	12	19.4	0.42	5.74	
12/18/2014	12	18.1	0.45	5.70	
12/22/2014	9	9.8	0.40	4.06	
12/25/2014	15	15.4	0.45	7.25	
			0.34	4.69	Average

Species：Murrah buffalo   Buffalo NO.：4

date(m/d/y）		Original semen		
	Room temperature	Quantity (ml)	Motility	Density(10^9/ml)
1/2/2014	20	12.8	0.34	6.33
1/6/2014	19	12.0	0.34	6.51
1/9/2014	13	8.7	0.34	8.55
1/13/2014	10	11.2	0.36	3.65
1/16/2014	8	9.5	0.35	5.48
1/20/2014	9	11.0	0.34	11.83
1/23/2014	6	12.7	0.36	3.02
1/27/2014	18	18.8	0.34	5.73
2/10/2014	9	13.1	0.34	2.27
2/13/2014	6	6.8	0.30	10.49
2/17/2014	11	8.1	0.35	2.71
2/20/2014	4	13.5	0.35	2.88
2/24/2014	13	10.0	0.34	11.24
2/27/2014	16.2	15.5	0.37	4.89
3/3/2014	13	10.9	0.37	5.94
3/6/2014	14	20.0	0.34	2.99
3/10/2014	11	12.2	0.32	7.70
3/13/2014	16	12.1	0.35	8.25
3/17/2014	15	12.7	0.35	11.35
3/20/2014	22	16.0	0.35	5.84

3/24/2014		15.8	0.30	9.87
3/27/2014	21	15.0	0.30	3.05
3/31/2014	24	14.4	0.30	9.04
4/7/2014	21	10.1	0.37	8.79
4/10/2014		11.8	0.36	3.89
4/14/2014	25	13.7	0.32	10.71
4/17/2014	25	15.6	0.35	6.69
4/21/2014	27	10.3	0.30	11.98
4/24/2014		10.7	0.35	8.26
4/28/2014	26	14.7	0.32	7.89
5/5/2014	20	17.5	0.35	3.26
5/8/2014	22	6.8	0.34	8.86
5/12/2014	27	12.5	0.38	10.05
5/15/2014	27	17.7	0.40	7.43
5/19/2014	27	13.2	0.40	6.13
5/22/2014	29	15.5	0.34	4.53
5/26/2014	31	12.3	0.39	8.02
5/29/2014	30	5.9	0.36	11.42
6/5/2014	30	11.4	0.36	6.86
6/9/2014	27	12.4	0.40	9.78
6/12/2014	26	11.0	0.35	6.74
6/16/2014	30	13.7	0.30	8.74
6/19/2014	29	13.3	0.40	4.56
6/23/2014	30	15.0	0.37	7.66
6/26/2014	30	13.3	0.36	4.04
6/30/2014	30	12.6	0.35	5.98
7/3/2014	30	14.2	0.20	4.54
7/7/2014	30	20.0	0.33	7.91
7/10/2014	30	12.6	0.36	9.52
7/14/2014	30	13.8	0.35	7.15
7/17/2014	31	12.0	0.35	6.20
7/21/2014	28	12.4	0.37	4.20
7/24/2014	30	15.6	0.36	6.21
7/27/2014	30	17.6	0.35	8.53
7/31/2014	31	12.9	0.36	4.59
8/4/2014	31	9.8	0.36	7.12
8/7/2014	30	15.9	0.40	3.36
8/11/2014	31	16.2	0.38	7.64
8/14/2014	24	15.4	0.36	8.46
9/11/2014	29	10.3	0.36	8.00

9/15/2014	31	13.1	0.35	7.45	
9/18/2014	29	13.8	0.38	1.99	
9/22/2014	29	8.2	0.38	2.69	
9/25/2014	28	14.6	0.44	7.79	
9/29/2014	28	9.7	0.37	5.80	
10/9/2014	22	11.1	0.37	4.23	
10/13/2014	24	12.2	0.37	5.38	
10/16/2014	22	14.8	0.35	9.27	
10/20/2014	27	19.7	0.36	5.31	
10/23/2014	23	14.3	0.37	9.19	
10/27/2014	26	16.6	0.36	4.58	
10/30/2014	27	14.2	0.42	6.73	
11/3/2014	16	11.0	0.46	5.64	
11/6/2014	20	11.7	0.36	7.94	
11/10/2014	18	7.7	0.35	7.52	
11/13/2014	17	11.4	0.36	5.49	
11/17/2014	17	16.3	0.30	10.09	
11/20/2014	18	7.2	0.36	5.16	
11/24/2014	19	12.4	0.37	9.45	
11/27/2014	18	9.1	0.36	8.50	
12/1/2014	19	11.9	0.37	7.66	
12/4/2014	14	13.4	0.36	9.98	
12/8/2014	12	8.4	0.35	15.38	
12/11/2014	13	14.8	0.36	8.93	
12/15/2014	12	13.5	0.35	4.80	
12/18/2014	12	12.2	0.35	8.08	
12/22/2014	9	7.9	0.35	6.84	
12/25/2014	15	5.2	0.68	10.58	
			0.36	7.02	Average

Species：Murrah buffalo   Buffalo NO.：5

date(m/d/y）	Room temperature	Original semen		
		Quantity (ml)	Motility	Density(10^9/ml)
1/2/2014	20	19.2	0.36	2.99
1/6/2014	19	17.5	0.34	3.33
1/9/2014	13	18.4	0.40	3.71
1/13/2014	10	12.5	0.50	8.07
1/16/2014	8	19.0	0.50	3.13

1/20/2014	9	13.4	0.55	6.65
1/23/2014	6	17.6	0.55	3.59
1/27/2014	18	15.4	0.50	3.63
2/10/2014	9	19.7	0.49	4.37
2/13/2014	6	17.5	0.49	3.30
2/17/2014	11	15.5	0.40	3.44
2/20/2014	4	14.6	0.40	4.21
2/24/2014	13	16.1	0.55	4.48
2/27/2014	16	16.5	0.55	3.29
3/6/2014	14	20.0	0.56	3.76
3/10/2014	11	12.9	0.53	4.03
4/7/2014	21	18.7	0.50	8.76
4/10/2014		14.0	0.50	2.64
4/14/2014	25	16.3	0.55	4.60
4/17/2014	25	15.9	0.50	4.56
4/21/2014	27	16.1	0.55	4.72
4/24/2014		13.7	0.50	3.99
4/28/2014	26	16.7	0.50	1.94
5/5/2014	20	23.6	0.53	6.04
5/8/2014	22	14.0	0.50	6.17
5/12/2014	27	13.9	0.56	8.30
5/15/2014	27	19.7	0.50	4.46
5/19/2014	27	14.7	0.55	8.06
5/22/2014	29	17.8	0.50	3.73
5/26/2014	31	19.9	0.56	6.21
5/29/2014	30	14.5	0.50	4.09
6/5/2014	30	11.9	0.55	4.61
6/16/2014	30	20.0	0.48	9.07
6/19/2014	29	15.2	0.50	4.49
6/23/2014	30	16.7	0.55	3.73
6/26/2014	30	15.3	0.55	3.73
6/30/2014	30	17.6	0.55	7.56
7/3/2014	30	15.9	0.58	6.07
7/7/2014	30	21.0	0.55	3.63
7/10/2014	30	20.1	0.56	3.31
7/14/2014	30	20.9	0.57	5.02
7/17/2014	31	16.5	0.55	3.06
7/21/2014	28	18.2	0.55	5.36
7/24/2014	30	19.3	0.55	3.73
7/28/2014	30	17.4	0.45	3.51

7/31/2014	31	20.0	0.50	3.52	
8/4/2014	31	20.5	0.55	3.42	
8/7/2014	30	16.5	0.45	4.59	
8/11/2014	31	18.7	0.50	5.06	
8/14/2014	24	18.1	0.54	3.16	
9/15/2014	31	15.9	0.56	4.54	
9/22/2014	29	12.7	0.55	6.09	
9/29/2014	28	14.4	0.45	7.31	
10/9/2014	22	13.4	0.55	5.54	
10/16/2014	22	15.1	0.58	7.72	
10/20/2014	27	14.3	0.50	5.72	
10/27/2014	26	16.4	0.55	10.52	
10/30/2014	27	15.3	0.50	5.89	
11/3/2014	16	14.0	0.55	2.44	
11/10/2014	18	12.2	0.50	6.80	
11/17/2014	17	16.0	0.55	1.28	
11/24/2014	19	11.0	0.55	8.25	
12/1/2014	19	13.4	0.55	10.68	
12/8/2014	12	11.2	0.55	7.71	
12/15/2014	12	16.5	0.55	3.46	
12/22/2014	9	15.3	0.55	5.73	
			0.52	5.01	Average

Species： Buffalo

Murrah buffalo NO.：6

date(m/d/y）		Original semen		
	Room temperature	Quantity (ml)	Motility	Density(10^9/ml)
1/2/2014	20	13.0	0.45	5.50
1/6/2014	19	10.5	0.42	5.33
1/9/2014	13	7.4	0.46	10.37
1/13/2014	10	9.5	0.42	7.94
1/16/2014	8	10.6	0.45	9.01
1/20/2014	9	10.4	0.47	7.90
1/23/2014	6	13.1	0.40	5.45
1/27/2014	18	9.7	0.48	6.32
2/10/2014	9	11.4	0.46	8.55
2/13/2014	6	9.1	0.42	6.30
2/17/2014	11	10.8	0.44	6.94
2/20/2014	4	11.0	0.46	7.09

2/24/2014	13	6.4	0.45	10.06
2/27/2014	16	8.6	0.46	4.68
3/3/2014	13	15.2	0.45	10.51
3/6/2014	14	12.4	0.47	73.37
3/10/2014	11	12.6	0.44	6.35
3/13/2014	16	13.7	0.44	7.27
3/17/2014	15	13.1	0.48	7.58
3/20/2014	22	10.2	0.45	7.58
3/24/2014		8.1	0.46	8.03
3/27/2014	21	10.2	0.46	10.58
3/31/2014	24	14.3	0.45	7.12
4/7/2014	21	19.0	0.48	7.53
4/10/2014		11.6	0.45	4.46
4/14/2014	25	8.4	0.45	20.33
4/17/2014	25	10.9	0.45	8.39
4/21/2014	27	12.1	0.45	4.41
4/24/2014		13.6	0.40	4.77
4/28/2014	26	9.2	0.45	3.73
5/5/2014	20	13.1	0.42	6.60
5/8/2014	22	10.6	0.45	7.86
5/12/2014	27	8.8	0.46	5.58
5/15/2014	27	13.3	0.45	6.64
5/19/2014	27	12.8	0.45	8.00
5/22/2014	29	11.1	0.40	5.53
5/26/2014	31	18.3	0.40	11.36
5/29/2014	30	12.3	0.42	6.62
6/5/2014	30	17.1	0.40	10.70
6/9/2014	27	13.8	0.40	7.79
6/12/2014	26	16.6	0.45	7.53
6/16/2014	30	11.6	0.45	6.07
6/19/2014	29	11.9	0.46	6.34
6/23/2014	30	13.8	0.47	7.49
6/26/2014	30	15.1	0.45	6.95
6/30/2014	30	12.7	0.46	6.77
7/3/2014	30	10.8	0.45	10.84
7/7/2014	30	9.5	0.45	6.81
7/10/2014	30	12.4	0.46	9.60
7/14/2014	30	15.8	0.45	6.61
7/17/2014	31	16.7	0.47	5.24
7/21/2014	28	17.3	0.47	4.92

7/24/2014	30	10.2	0.45	6.22	
7/28/2014	30	14.9	0.45	5.07	
7/31/2014	31	8.8	0.46	8.63	
8/4/2014	31	15.5	0.45	8.42	
8/7/2014	30	11.1	0.45	7.97	
8/11/2014	31	13.2	0.46	9.17	
8/14/2014	24	8.3	0.47	7.81	
9/11/2014	29	24.7	0.47	5.00	
9/15/2014	31	13.4	0.46	5.10	
9/18/2014	29	8.6	0.45	6.55	
9/22/2014	29	14.8	0.40	9.86	
9/25/2014	28	11.4	0.46	6.00	
9/29/2014	28	10.7	0.45	5.68	
10/9/2014	22	13.8	0.40	7.47	
10/13/2014	24	17.1	0.47	9.14	
10/16/2014	22	9.1	0.48	7.32	
10/20/2014	27	15.2	0.45	5.66	
10/23/2014	23	12.5	0.40	8.25	
10/27/2014	26	11.7	0.45	7.90	
10/30/2014	27	9.7	0.45	6.76	
11/3/2014	16	10.8	0.46	6.52	
11/6/2014	20	5.9	0.46	8.13	
11/10/2014	18	11.7	0.42	9.83	
11/13/2014	17	9.6	0.46	6.57	
11/17/2014	17	9.6	0.44	7.35	
11/20/2014	18	121.6	0.40	8.75	
11/24/2014	19	10.2	0.46	8.28	
11/27/2014	18	9.2	0.45	7.96	
12/1/2014	19	13.7	0.48	7.22	
12/4/2014	14	12.7	0.45	9.99	
12/8/2014	12	9.1	0.45	11.58	
12/11/2014	13	15.5	0.43	9.96	
12/15/2014	12	10.2	0.40	5.36	
12/18/2014	12	14.3	0.45	5.93	
12/22/2014	9	12.6	0.42	8.43	
12/25/2014	15	13.8	0.45	6.44	
			0.45	8.43	Average

Species：Murrah buffalo   Buffalo NO.：7

date(m/d/y）	Room temperature	Original semen		
		Quantity (ml)	Motility	Density(10^9/ml)
1/7/2014	11	9.1	0.4	9.78
1/10/2014	13	5.5	0.4	10.75
1/14/2014	10	10.5	0.45	7.11
1/17/2014	16	6.3	0.45	7.38
1/21/2014	18	8.4	0.4	8.56
1/24/2014	18	6.3	0.5	6.52
1/28/2014	16	7.4	0.2	10.39
1/31/2014	20	8.6	0.45	8.73
2/4/2014	22	7	0.5	11.13
2/18/2014	20		0.1	
2/21/2014	14	8.8	0.45	8.59
2/25/2014	18	6.1	0.4	11.02
3/4/2014	18	8.5	0.3	11.47
3/7/2014	19	8.8	0.45	7.29
3/11/2014	22	3.97	0.53	12.72
3/14/2014	22	8.2	0.4	5.9
3/18/2014	22	9.1	0.3	7.54
3/21/2014	25	12.1	0.45	3.94
3/25/2014	27	8	0.45	8.12
3/28/2014	22	6.6	0.5	8.2
4/1/2014	22	7.3	0.53	8.28
4/8/2014	21	9.4	0.5	7.74
4/11/2014	20	4.4	0.45	6.87
4/15/2014	20	13.4	0.5	5.76
4/18/2014	20	3.4	0.44	9.86
4/22/2014	22	10.7	0.4	9.57
4/25/2014	26	6.1	0.54	9.27
5/2/2014	25	8.6	0.52	10.76
5/6/2014	24	6.8	0.48	12.01
5/9/2014	26	9	0.52	7.91
5/13/2014	26	7.2	0.52	8.66
5/16/2014	28	3.9	0.5	10.29
5/20/2014	28	6.9	0.47	11.72
5/23/2014	28	10.4	0.53	4.41

5/27/2014	28	8.6	0.5	11.55
5/30/2014	27	4.4	0.54	7.24
6/3/2014	28	5.9	0.54	11.71
6/17/2014	28	8.8	0.56	14.7
6/20/2014	28	8.2	0.55	9.77
7/4/2014	27.5	12.3	0.5	8.82
7/8/2014	28	9.2	0.52	10.29
7/11/2014	28	4.5	0.48	11.37
7/15/2014	28	10.6	0.53	10.22
7/22/2014	28	13.3	0.52	8.75
7/25/2014	28	8.4	0.45	10.48
7/29/2014	27	6.7	0.45	9.87
8/1/2014	28	12	0.45	4.63
8/26/2014	28	11.5	0.45	9.42
8/29/2014	28	13.2	0.54	6.27
9/2/2014	28	9.3	0.53	8.37
9/5/2014	26	12.2	0.46	5.11
9/9/2014	28	8.7	0.47	9.37
9/12/2014	28	2.4	0.48	11.33
9/16/2014	28	8	0.53	10.49
9/23/2014	29	9.7	0.53	11.95
9/26/2014	25	7.1	0.5	10.41
9/30/2014	28	5.9	0.53	17.41
10/10/2014	26	12	0.48	10
10/14/2014	27	8.2	0.55	9.06
10/17/2014	26	6	0.52	10.57
10/21/2014	22	7.4	0.54	9.92
10/24/2014	24	6.8	0.54	9.6
10/28/2014	24	6.6	0.54	10.91
10/31/2014	24	10.6	0.53	7.72
11/4/2014	24	12.8	0.47	6.61
11/7/2014	26	6	0.5	12.92
11/14/2014	22	7.7	0.45	10.85
11/18/2014	22	7	0.45	10.95
11/21/2014	20	9.5	0.5	6.69
11/25/2014	25	8.7	0.5	8.8
11/28/2014	25	6.1	0.49	6.45
12/2/2014	18	9.4	0.52	7.38
12/5/2014	20	7.5	0.44	7.03
12/9/2014	20	7.8	0.44	8.23

12/12/2014	20	10.1	0.52	7.31	
12/16/2014	9	14.6	0.5	6.22	
12/19/2014	6	6.4	0.53	6.85	
12/23/2014	6	7.5	0.5	7.65	
12/26/2014	7	5.9	0.3	9.21	
12/30/2014	8	8.9	0.1	4.86	
			0.47	9.03	Average

Species：Murrah buffalo　　Buffalo NO.：8

date(m/d/y）	Room temperature	Original semen Quantity (ml)	Motility	Density(10^9/ml)
1/2/2014	20	8.2	0.20	5.48
1/6/2014	19	11.3	0.20	7.03
1/9/2014	13	14.0	0.20	6.70
1/13/2014	10	14.7	0.30	6.48
1/16/2014	8	5.0	0.20	10.73
1/20/2014	9	10.0	0.40	8.51
1/23/2014	6	10.5	0.30	8.19
1/27/2014	18	12.9	0.20	6.69
2/10/2014	9	18.5	0.35	6.90
2/13/2014	6	15.0	0.30	7.41
2/17/2014	11	8.3	0.30	2.79
2/20/2014	4	10.5	0.30	3.46
2/24/2014	13	13.5	0.20	7.78
2/27/2014	16	11.3	0.10	4.03
3/3/2014	13	9.5	0.45	5.43
3/6/2014	14	10.6	0.30	6.04
3/10/2014	11	8.5	0.30	5.82
3/13/2014	16	13.6	0.30	3.46
3/17/2014	15	9.9	0.30	8.96
3/20/2014	22	11.1	0.10	1.87
3/24/2014		10.0	0.30	3.10
3/27/2014	21	9.0	0.20	3.91
3/31/2014	24	10.0	0.10	4.21
4/7/2014	21	12.8	0.38	8.76
4/10/2014		12.8	0.40	2.46
4/14/2014	25	7.8	0.20	6.85
4/17/2014	25	9.4	0.10	9.14

4/21/2014	27	10.6	0.35	2.07
4/24/2014		3.6	0.40	2.00
4/28/2014	26	12.1	0.35	0.71
5/5/2014	20	9.6	0.50	4.18
5/12/2014	27	12.4	0.30	6.22
5/15/2014	27	9.4	0.30	6.96
5/22/2014	29	14.0	0.30	6.85
6/5/2014	30	8.9	0.30	10.58
6/9/2014	27	14.5	0.38	10.80
6/12/2014	26	5.7	0.42	12.48
6/16/2014	30	9.2	0.40	10.28
6/19/2014	29	10.0	0.38	6.42
6/23/2014	30	10.5	0.40	8.83
6/26/2014	30	7.3	0.47	8.40
6/30/2014	30	8.5	0.44	9.04
7/3/2014	30	7.4	0.44	7.92
7/7/2014	30	7.9	0.42	9.22
7/10/2014	30	10.1	0.42	5.69
7/14/2014	30	8.2	0.45	9.35
7/17/2014	31	5.2	0.47	8.96
7/21/2014	28	11.0	0.40	11.48
7/24/2014	30	7.4	0.45	3.90
7/28/2014	30	8.5	0.46	8.35
7/31/2014	31	7.1	0.46	7.85
8/4/2014	31	11.9	0.45	4.50
8/11/2014	31	9.2	0.47	11.48
8/14/2014	24	11.4	0.47	7.78
9/11/2014	29	11.7	0.40	8.80
9/15/2014	31	9.7	0.46	7.23
9/18/2014	29	9.4	0.55	6.41
9/22/2014	29	8.1	0.45	10.21
9/25/2014	28	4.2	0.42	5.96
9/29/2014	28	3.9	0.46	8.86
10/9/2014	22	7.2	0.45	13.43
10/13/2014	24	5.9	0.45	12.20
10/16/2014	22	7.6	0.47	9.37
10/20/2014	27	8.8	0.45	7.33
10/23/2014	23	10.0	0.46	4.90
10/27/2014	26	10.5	0.45	6.43
10/30/2014	27	8.1	0.47	10.53

11/3/2014	16	10.1	0.40	11.62	
11/6/2014	20	8.1	0.35	3.45	
11/10/2014	18	8.3	0.42	3.67	
11/13/2014	17	6.3	0.40	9.94	
11/17/2014	17	5.1	0.35	2.97	
11/24/2014	19	11.5	0.30	8.94	
11/27/2014	18	5.6	0.45	17.44	
12/1/2014	19	7.6	0.40	10.52	
12/8/2014	12	7.5	0.40	12.77	
12/15/2014	12	8.0	0.10	8.33	
12/18/2014	12	2.0	0.20	17.28	
12/22/2014	9	6.5	0.20	10.81	
12/25/2014	15	4.0	0.10		
			0.35	7.52	Average

Species：Murrah buffalo　　Buffalo NO.：9

date(m/d/y）		Original semen		
	Room temperature	Quantity (ml)	Motility	Density(10^9/ml)
1/2/2014	20	5.3	0.45	10.12
1/6/2014	19	7.9	0.40	9.57
1/9/2014	13	7.8	0.40	12.44
1/13/2014	10	6.0	0.42	12.99
1/20/2014	9	6.2	0.42	17.08
1/23/2014	6	5.7	0.40	5.51
1/27/2014	18	6.4	0.45	9.07
2/10/2014	9	9.4	0.46	9.51
2/13/2014	6	7.6	0.46	10.92
2/17/2014	11	6.3	0.40	11.69
2/20/2014	4	8.7	0.45	9.00
2/24/2014	13	10.2	0.40	11.93
2/27/2014	16	3.6	0.40	9.13
3/3/2014	13	6.3	0.40	10.06
3/6/2014	14	4.3	0.40	11.37
3/10/2014	11	5.5	0.48	7.84
3/17/2014	15	6.1	0.30	11.47
3/20/2014	22	5.1	0.40	16.53
3/24/2014		6.4	0.45	11.81
3/27/2014	21	8.0	0.45	12.58

4/7/2014	21	7.4	0.45	12.29
4/14/2014	25	7.0	0.43	14.75
4/24/2014		7.9	0.40	7.62
4/28/2014	26	9.0	0.46	10.41
5/5/2014	20	9.4	0.43	13.07
5/8/2014	22	6.8	0.46	12.54
5/12/2014	27	7.5	0.45	16.78
5/15/2014	27	8.8	0.46	12.53
5/19/2014	27	7.7	0.46	17.52
5/22/2014	29	9.5	0.43	7.91
5/26/2014	31	9.5	0.46	11.87
5/29/2014	30	8.1	0.46	10.94
6/5/2014	30	8.1	0.44	10.84
6/9/2014	27	11.3	0.40	12.86
6/12/2014	26	6.1	0.43	14.94
6/19/2014	29	6.4	0.44	9.65
6/23/2014	30	6.4	0.45	14.52
6/30/2014	30	5.6	0.43	11.82
7/3/2014	30	7.4	0.47	8.09
7/7/2014	30	6.7	0.45	9.16
7/10/2014	30	5.9	0.45	8.07
7/14/2014	30	7.7	0.44	7.51
7/17/2014	31	7.9	0.44	8.39
7/21/2014	28	6.5	0.42	8.47
7/24/2014	30	6.4	0.43	8.45
7/28/2014	30	10.3	0.45	7.65
7/31/2014	31	6.9	0.46	8.82
8/4/2014	31	5.0	0.45	6.91
8/7/2014	30	7.5	0.47	8.00
8/11/2014	31	8.4	0.47	10.62
8/14/2014	24	7.6	0.45	7.90
9/11/2014	29	11.1	0.43	10.98
9/15/2014	31	8.7	0.45	11.77
9/18/2014	29	9.2	0.48	10.00
9/22/2014	29	6.0	0.48	11.84
9/25/2014	28	10.0	0.48	7.24
9/29/2014	28	5.9	0.48	10.85
10/9/2014	22	7.9	0.43	12.76
10/13/2014	24	6.6	0.48	9.83
10/16/2014	22	8.5	0.40	11.43

10/20/2014	27	8.2	0.46	13.70	
10/23/2014	23	505.0	0.48	11.41	
10/27/2014	26	6.6	0.48	10.23	
10/30/2014	27	6.9	0.46	10.41	
11/3/2014	16	22.8	0.10	4.03	
11/6/2014	20	7.1	0.36	7.82	
11/10/2014	18	5.6	0.30	7.67	
11/13/2014	17	7.1	0.36	12.03	
11/17/2014	17	7.4	0.30	7.76	
12/11/2014	13	5.1	0.40	16.65	
12/15/2014	12	7.9	0.40	14.56	
12/18/2014	12	7.5	0.45	11.60	
12/22/2014	9	7.5	0.40	12.21	
12/25/2014	15	6.9	0.45	16.71	
			0.43	10.88	Average

Buffalo

Species：Murrah buffalo NO.：10

date(m/d/y）		Original semen		
	Room temperature	Quantity (ml)	Motility	Density(10^9/ml)
1/7/2014	11	17.8	0.45	2.1
1/10/2014	13	17.9	0.45	2.99
1/14/2014	10	19.8	0.47	3.85
1/17/2014	16	13.8	0.4	5.53
1/21/2014	18	19.3	0.42	2.79
1/24/2014	18	8.2	0.4	2.01
1/28/2014	16	14.1	0.45	5.62
1/31/2014	20	12.8	0.45	6.7
2/4/2014	22	18.9	0.4	3.87
2/18/2014	21	19	0.45	4.98
2/21/2014	14	12.7	0.45	3.61
2/25/2014	18	15.1	0.4	4.29
2/28/2014	22	15.7	0.45	4.87
3/4/2014	18	13.8	0.4	4.46
3/7/2014	19	3.1	0.45	3.01
3/11/2014	22	17.4	0.45	3.92
3/14/2014	22	15.8	0.46	3.74
3/18/2014	22	18.7	0.4	5.01
3/21/2014	25	18.3	0.47	3.65

3/25/2014	27	13	0.45	4.73
3/28/2014	22	17.1	0.4	4.52
4/1/2014	22	21.1	0.44	3.35
4/8/2014	21	18.9	0.45	3.61
4/11/2014	20	14.5	0.4	3.74
4/15/2014	20	15.8	0.43	5.81
4/18/2014	20	14.1	0.43	3.46
4/22/2014	22	12.6	0.4	4.54
4/25/2014	26	16	0.4	2.47
5/2/2014	25	10.2	0.46	8.68
5/6/2014	24	11.8	0.47	5.13
5/9/2014	26	19	0.45	3.86
5/13/2014	26	19.7	0.44	4.37
5/16/2014	28	13.8	0.4	4.5
5/20/2014	28	10.8	0.45	8.85
5/23/2014	28	17.1	0.45	4.44
5/27/2014	28	11.5	0.45	5.65
5/30/2014	27	16.2	0.45	4.33
6/3/2014	28	8.8	0.42	6.8
6/6/2014	28	15.9	0.43	4.15
6/13/2014	28	17.6	0.45	4.51
6/17/2014	28	10.7	0.47	9.14
6/20/2014	28	17.8	0.45	4.54
6/24/2014	28	11.5	0.45	6.33
7/1/2014	28	17.3	0.45	7.02
7/4/2014	27.5	7.7	0.42	6.44
7/8/2014	28	9.7	0.44	10.9
7/11/2014	28	13.6	0.44	6.07
7/15/2014	28	10.1	0.42	5.71
7/18/2014	28	9.7	0.4	4.82
7/23/2014	28	18	0.45	4.48
7/25/2014	28	11.5	0.48	5.71
7/29/2014	27	14.6	0.45	4.39
8/1/2014	28	18.6	0.44	4.54
8/26/2014	28	13.6	0.44	6.53
8/29/2014	28	12.5	0.4	4.39
9/2/2014	28	14.2	0.42	5.03
9/5/2014	26	11.9	0.45	4.39
9/9/2014	28	13.8	0.45	4.82
9/12/2014	28	14	0.44	4.83

9/16/2014	28	10.9	0.43	6.59	
9/23/2014	29	13.9	0.45	9.06	
9/26/2014	25	15.4	0.48	3.81	
9/30/2014	28	10	0.45	3.97	
10/10/2014	26	17	0.47	4	
10/14/2014	27	10.5	0.45	7.16	
10/17/2014	26	17.76	0.4	4.8	
10/21/2014	22	10.9	0.43	7.43	
10/24/2014	24	14.2	0.42	10.04	
10/28/2014	24	12.2	0.4	5.33	
10/31/2014	24	14.9	0.41	4.34	
11/4/2014	24	10.4	0.4	5.1	
11/7/2014	26	16.2	0.45	5.46	
11/14/2014	22	11.6	0.45	5.45	
11/18/2014	22	14.4	0.48	7.18	
11/21/2014	20	16	0.4	5.34	
11/25/2014	25	14	0.4	4.88	
11/28/2014	25	7.8	0.46	7.12	
12/2/2014	18	8.9	0.43	6.25	
12/5/2014	20	10.9	0.44	6.63	
12/9/2014	20	13.3	0.42	5.25	
12/12/2014	20	10.4	0.42	5.31	
12/16/2014	9	15.8	0.44	4.64	
12/19/2014	6	9.8	0.46	7.76	
12/23/2014	6	9.7	0.46	9.8	
12/26/2014	7	7.6	0.46	7.75	
12/30/2014	8	6	0.47	9.36	
			0.44	5.33	Average

- List of Abbreviations

Abbreviation	• Full title
PHB	Prohibitin
CAPZB	F-actin-capping protein subunit beta
TEKT2	Tektin-2
HSP70	Heat shock 70 kDa protein

PRDX6	DNA binding domain
VAMP4	Vesicle-associated membrane protein 4
ODF	Outer dense fiber protein
AKAP4	A-kinase anchor protein 4
DNA	Deoxyribonucleic acid
RNA	Ribonuclease
mRA	Message RNA
CDS	Coding sequence
CIB1	calcium- and integrin-binding protein 1
ACTB	β-Actin
SABP	Salicylic acid receptor protein
Western-blot	Western-blot
PCR	Polymerase chain reaction
ORF	Open reading frame
QRT-PCR	Quantitive real time PCR
ANXA5	annexin a5
DNAII	The axonemal dynein arms medium chain II
DNAH5	The axonemal dynein heavy chain 5 arm
DNAH11	The axonemal dynein arms 11
PRDX6	Peroxiredoxin-6
PRM2	Recombinant Protamine 2
GnRHR	gonadotropin-releasing hormone receptor
APES	(3- amino propyl) triethoxy sila ne
PFA	paraformaldehyde

SmCP	Cysteine protease
RIPA	RIPA Lysis Buffer（Radio
PMSF	Phenylmethanesulfonyl fluoride
SEPT4	Septin 4
GAPDHS	glyceraldehyde-3-phosphate dehydrogenase
IDH-α	isocitrate dehydrogenases
ODF	Outer dense fiber protein
NHA1	Kandelia candel-sourced sodium-hydrogen t
Prm1	protamine1
ENOS	endothelial nitric oxide synthase
NNOS	neuronal nitric oxide synthase

Addtional Data Table S1

Murrah buffalo PHB encoding sequence(Shanghai Sangon sequencing)

CGCAATCGGCAGTGCAGCTTGCATGCCTGCAGGTCGACGATTCCTTCACTT
TAAGCCAATCATGAAGTTTCACAGTGATTTCTGGGGCAGGAGAAGG
AAGGTGGTGCTGAGCGACTTCGGGGCTGTGGTCCAGCTGGCCCAGG
AGGTGTGGGCCTCACTGGGGCAGCTGGAGGAGCACCGACTGCCCGG
TGGGCAGGTAGGTGATGTTCCGGGAGCGCGACAGCTGGTACGCGAT
GTCTTCCGCGGCCTCCAGCTTGCGCAGCTCGATCAGGCCGTCGCCTG
CAGTGGCGAGCGAGTTGGCAATTAACTCCGCCGCCTTGGAGTCGCCC

TCTGCGGAGATGATGGCTGCCTTCTTCTGCTGCTCAGCCTTTTCCACC
ACAAATCTGGCCCTCTCTGCTTCCTGCTGAGCCACCTGTTTGGCTTCC
ACCGCTTCTGTGAACTCCTTCCCAAAGGTCAGATGCGTCAAGGATAC
GTCATCCAGGATGAGCCCAAAGGTCGCTGCTCGCTCTGTGAGGTCAT
CGCTCACCTGTCTGGAGACCAGCTCTCTCTGGGTGATCAGTTCTCCA
GCATCAAAGCGAGCCACCACGGACTTGAGGATCTCTGTAGTGATGG
ATGGCAGCACGCGCTCGTCGTAGTCCTCTCCGATGCTGGTGAAGATG
CGAGGAAGCTGACTAGCAACCGGCCGGAAGAGGATGCGCAGCGTGA
TGTTGACATTCTGTAAATCTTTGCTACCAGTGATTACTGGCACATTAC
GTGGTCGAGAGCGGCAGTCAAAGATAATTGGTTTCTGTACCCAAGGG
ATGAGGAAGTGAGTCCCTTCTCCTACGACAATGTCCTGCACTCCCCG
GAACCGGTCAAAGATGACAGCTCTGTGCCCAGCATCCACATTATACA
AGGCAGAGTTCACCACGCCTCCTGCAACCGCTAAGGCAAGACCAAA
CTTGCCAATGGACTCAAACACTTTGGCAGCCATGTCTCCTTTGGCTG
GACAATCTCTAGAGGATCCCCGGGTACCGAGCTCGAATCGTAATCAT
GTCATTGCCCC

Murrah buffalo CAPZB encoding sequence (Shanghai Sangon sequencing)

GGGGGATCGGCCCCGCTGCATTGTAATACGACTCACTATAGGGCGAATTG
GGCCCTCTAGATGCATGCTCGAGCGGCCGCCTTTGTGATGGATATCT
GCAGAATTGCCCTTCTCCGAGGCCAGCAGACGGCCCGGACACGATG
CACCCCTGCAGACGCAGCCTCCCTTTCCCCCTCAACTGTCAACTCGTG
AAGGTTGGAACTGCAGATTATGGAGGTGCCTCGGACCAGAGTGATC
AGCAGCTGGACTGTGCCTTGGACTTGATGAGGCGCCTGCCTCCACAG
CAAATCGAGAAAAACCTCAGTGACCTGATTGATCTGGTGCCCAGCCT
GTGTGAAGACCTCCTGTCCTCGGTTGACCAGCCACTGAAAATCGCCA
GAGACAAGGTGGTGGGGAAGGACTACCTCTTGTGTGACTACAACAG
GGACGGGGACTCCTACAGGTCACCATGGAGTAACAAGTATGACCCT
CCCCTGGAAGACGGGGCCATGCCTTCTGCTCGCCTGAGGAAGCTGGA
GGTGGAAGCCAACAACGCCTTTGACCAGTACCGAGACCTGTATTTTG
AAGGTGGTGTCTCATCTGTCTACCTCTGGGATCTGGATCATGGTTTTG
CTGGAGTGATCCTCATAAAGAAGGCTGGAGATGGATCAAAGAAGAT
CAAAGGCTGTTGGGACTCCATCCACGTGGTGGAGGTGCAGGAGAAG
TCCAGTGGTCGCACCGCCCATTACAAGCTGACCTCCACGGTGATGCT
GTGGCTGCAAACCAACAAATCCGGCTCTGGCACCATGAACCTCGGA
GGCAGCCTCACCAGACAGATGGAGAAGGATGAGACCGTGAGCGACT
GCTCTCCACACATAGCCAACATCGGGCGCCTGGTCGAGGACATGGA
GAATAAAATCAGAAGTACGCTGAACGAGATCTACTTTGGGAAGACA
AAGGACATCGTTAATGGGCTGAGGTCTGTGCAGACTTTTGCAGACAA
ATCAAAACAAGAAGCTCTTAAGAACGACCTGGTGGAGGCTTTGAAG
AGAAAGCAGCAGTGTTAAAAGCCTCTGCTTCCCACTGACCGAAGGG
CAATTCCAGCACACTGGCGGCCGTTACTAGTGGATCCCCCT

Murrah buffalo TEKT2 encoding sequence(Shanghai Sangon sequencing)

GCCTTTCAGGGCAGTTGATTGGTAATACGACTCACTATAGGGCGAATTGG
GCCCTCTAGATGCATGCTCGAGCGGCCGCCAGTGTGATGGATATCTG
CAGAATTGCCCTTGCGGAAGGAGAGTTAGGTGTCCTGGAACAGGCCT
GGGGGCCTGACCCTTCAGGACCTGCGCCATGGCCACGCTGAGCGTCA
AGCCCAGTCCACGCTTCCGGTTGCCGGACTGGCAGACCAACAGCTAC
CTGTTGTCCACCAACGCCGAGCGCCAGCGAGATGCCTCACACCAGAT
CCGCCAGGAGGCCCGGGTCCTCCGCAACGAGACCAACAACCAGACC
ATTTGGGATGAACATGACAATAGGACGCGGCTGGCAGAGAGGATTG
ATACTGTCAGCCGATGGAAGGAGATGCTGGACAAGTGTTTGACGGA
TTTAGATGCTGAGATCGACGCCCTGGCACAGATGAAAGAGTCAGCG
GAGCAAAACCTGCAGGCCAAGAACCTGCCTCTGGATGTGGCAATTG
AATGCCTGACCCTGCGGGAGAGTCGGCGTGACATTGATGTGGTGAA
GGACCCCGTGGAGGAGGAGCTGCACAAAGAGGTGGAGGTCATTGAG
GCCACCAAGAAGGCCTTGCAACAGAAGATCAGCCAGGCCTTTGAGA
AGCTCTTCCTCCTGCAGGAAGCCCGACAGCGGCTCAACTCTGACCAT
CGTGGCAAAATGGAGACACTGGACATTGACAGAGGCTGCCTCTCTCT
CAAACTCACGTCCCCGAACATCTCTCTGAAGATCAATCCCACACGTG
TGCCCAATAGCTCCACCTCACTCCAGCAGTGGGATGACTTCAGTCGG
TTCAACAAGGACCAGGGAGAGGCTGAGATGAAAAAGGCCACTGAGC
TGAGGGAGGCCATCGCCCTCACCATTGCTGAGACCAACAACGAGCT
GGAAGCCCAGAGGGTTGCCACGGAATTTGCCTTCAGGAAGCGGCTG
CGGGAGATGGAGAAACTGTACAGTGAGCTCAAGTGGCAAGAGAAGA
ATACCTTGGAGGAGATTGCCGAGCTGCATGAGGACATCCGGCACCTG
GAGGAGGACCTGCGCAGAAAGTTACAGAACCTGAAGCTGTGCCACA
CACGGCTAGAGGCCAGGACCTACCGGCCCAACGTGGAACTCTGCAG
GGACCAGGCACAGTACGGCCTCACCGACGAGGTTCACCAGTTAGAG
GCAACCATTGCCGCCCTGAAGCAGAAGCTGGCTCAGGCACAGGACA
CTCTGGATGCCCTGTACAAGCATCTGGCCCGGCTGCAGGCTGACATC
GCTTGCAAGGCCAACTCCATGCTGTTGGACACCAAGTGCACGGACAC
CCGTCGGAAGCTGACCGTGCCTGCTGAGAAGTTTGTGCCTGAGGTGG
ACACCTTCAACCGCACCACAAACCGCACCCTGAGTCCTCTCAAAACC
TGCCAGCTGGAGCTGGCCTAGGCAGGGGGCTGAGGGAGGAGAGGAA
GGCTGGTTGAAAATGGAAGGGATGGGGGGAGCGGAGAGAATGAATC
TAATAAAGGTCGGGATTCTCAGTAAGGGCAATTCCAGCACACTGGCG
GCCGTTACTAGTGGATCCGAGCTCGGTACCAAGCTTGGCGTAATAAA
TGTTCAATTTGTTCC
